# Fuzzy bipolar soft semiprime ideals in ordered semigroups

**DOI:** 10.1016/j.heliyon.2021.e06618

**Published:** 2021-04-09

**Authors:** Aziz-ul-Hakim, H. Khan, I. Ahmad, A. Khan

**Affiliations:** a Department of Mathematics, University of Malakand, Chakdara Dir(L), Pakistan; b Department of Mathematics, Abdul Wali Khan University Mardan, Pakistan

**Keywords:** Ordered semigroup, FBS left ideal, FBS right ideal, FB ideal, FBS semiprime ideal, FBS prime ideal

## Abstract

In this paper, we introduce fuzzy bipolar soft semiprimality in the structure of ordered semigroups and investigate some properties of the concept. Moreover, ordered semigroups and their some classes are characterized by means of fuzzy bipolar soft semiprimality. Finally, the Cartesian product of fuzzy bipolar soft semiprime (resp., prime) ideals over ordered semigroups is examined. Some of the ideas are supported by apt examples.

## Introduction

The Zadeh's concept of fuzzy set [1] has proved to be a big boom in the modern world of science and technology. The notion is so much innovative, crucial and ingenious that, since its emergence in 1965, it has touched almost each and every area of research exploration and analysis across the world. At present, the study of fuzzy set theory is in a rapid progress with multiple research dimensions. This remarkable concept was extended to various areas of mathematics by researchers. Chang [2] applied the idea to general topology and transformed some of the basic concepts of topological spaces to fuzzy topological spaces. Rosenfeld [3] is the first who initiated the study of the concept on abstract algebra by defining fuzzy subgroupoid (resp., subgroup). Similarly, Kuroki [4] extended the same concept to semigroups and initiated the study of fuzzy semigroup theory. Liu [5] studied the concept of fuzzy set on ring theory and, among others, introduced fuzzy (left, right) ideals of rings. In the same way, Kehayopulu and Tsingelis [6] took the lead in extending the concept to ordered semigroup theory. They [7], [8], [9] studied, among others, fuzzy ideal theory in ordered semigroups and introduced the concept of fuzzy left (resp., right, two-sided, bi-, interior, quasi-) ideals in ordered semigroups and examined their related properties. Moreover, they characterized various classes of ordered semigroups in terms of these ideals. Jirojkul and Chinram [10] defined fuzzy quasi-ideal subsets and fuzzy quasi-filters in ordered semigroups and characterized ordered semigroups by these notions. Shabir and Iqbal [11] introduced bipolar fuzzy left (resp., right, bi-) ideals in ordered semigroups and characterized their various classes by the properties of these ideals. Similarly, Xie and Tang [12], [13], Shabir and Khan [14], [15], Davvaz and Khan [16], and F. M. Khan et al. [17], [18] studied ordered semigroups by means of fuzzy set theory. In the same fashion, many other researchers [19], [20], [21], [22], [23] contributed a lot to fuzzy ideal theory in ordered semigroups through various dimensions. On the other hand, Hayat et al. [24] studied bipolar fuzzy BRK-ideals in BRK-algebras and discussed their related properties. Karaaslan et al. [25] applied the notion of bipolar soft sets to group theory, and thus defined bipolar soft groups and examined some of their related properties. In [26], the notions of bipolar anti fuzzy h-ideals and bipolar anti fuzzy interior h-ideals in hemirings were introduced and some properties of these concepts were investigated. In [27], the authors introduced some new operations on type-2 soft sets and examined related properties. Sana et al. [28] introduced and studied the concepts of possibility fuzzy soft ideals and possibility fuzzy soft interior ideals in ordered semigroups. In [29], the authors applied soft set, based on acceptable and satisfactory levels, to design concept evaluation techniques.

In 1982, Kuroki [30] initiated and examined the concept of fuzzy semiprimality in semigroups (without order), whereas, in ordered semigroup theory, Shabir and Khan [31] studied the concept of semiprime fuzzy quasi-ideals. Likewise, Kehayopulu and Tsingelis [7] characterized fuzzy left (resp., right) semiprime ideals of ordered semigroups. Muhiuddin et al. [32] further studied fuzzy semiprime subsets in ordered semigroups. It is worth mentioning that Naz and Shabir [33] introduced the concept of fuzzy bipolar soft set which is an awesome blending of fuzzy and soft set theories. They examined the algebraic properties of the notion and studied its application in decision making problems. Since the concept of fuzzy bipolar soft set is a novel one, thus, in the present paper, we study semiprimality in ordered semigroups in terms of this structure, and hence the notion of fuzzy bipolar soft semiprimality is introduced. In addition, some properties of the concept are investigated on left (resp., right, intra-, completely) regular and Archimedean ordered semigroups. Besides, the Cartesian product of fuzzy bipolar soft semiprime (resp., prime) ideals over ordered semigroups is studied.

## 1 Preliminaries

Here, we state some definitions that are helpful in comprehending the content of the paper. For studying the applications of FBS set theory and further details, the readers may resort to [33]. An ordered semigroup (S,⋅,≤) is a set *S* where (S,⋅) is a semigroup and (S,≤) is a partially ordered set (poset) such that the order relation “≤” is compatible with the binary operation of multiplication “⋅”. Moreover, for a nonempty subset *P* of *S*, we say that *P* is a subsemigroup of *S* if and only if: (i) PP⊆P, and (ii) if a∈P and S∋b≤a then b∈P. Similarly, if *P* be a nonempty subset of *S*, then we denote by (P] the subset of *S* defined as follows:

(P]={s∈S|s≤pfor some p∈P}.

Let's recall that Naz and Shabir [33] presented the concept of fuzzy bipolar soft set which is a hybrid structure of fuzzy and soft set theories. Moreover, it possesses an intrinsic property of bipolarity. In the following, the concept is redefined so that ordered semigroups may be studied more conveniently in terms of fuzzy bipolar soft set theory.

Definition 1 Assume *S* be an initial universe set, F(S) the collection of all fuzzy subsets of *S* and *E* a set of parameters. For A⊆E, let f:A→E be an injective function. Then, a fuzzy bipolar soft (FBS) set $\lambda_{A}$ over *S* is an object of the form

$$\lambda_{A}=(\overset{+}{\lambda },\overset{-}{\lambda },A),$$

where $\overset{+}{\lambda }:A\to F(S)$ and $\overset{-}{\lambda }:f(A)\to F(S)$ are set-valued functions such that the condition

$$0\leq \overset{+}{\lambda }(\varepsilon )(x)+\overset{-}{\lambda }(f(\varepsilon ))(x)\leq 1$$

holds, for all ε∈A and x∈S.

**Note.** In what follows, the universe set *S* always represents an ordered semigroup. Further, instead of $\left(\overset{+}{\lambda },\overset{-}{\lambda },A\right)$, we write $\left(\overset{+}{\lambda },\overset{-}{\lambda }\right)$ for the sake of convenience.

Definition 2 Let $\lambda_{A}$ be an FBS set over *S* satisfying the condition $x\leq y\Rightarrow \overset{+}{\lambda }(\varepsilon )(x)\geq \overset{+}{\lambda }(\varepsilon )(y)$, $\overset{-}{\lambda }(\varepsilon )(x)\leq \overset{-}{\lambda }(\varepsilon )(y)$ for all ε∈A, where x,y∈S. Then, it is called an FBS ordered semigroup over *S* if and only if $\overset{+}{\lambda }(\varepsilon )(xy)\geq \min(\overset{+}{\lambda }(\varepsilon )(x),\overset{+}{\lambda }(\varepsilon )(y))$ and $\overset{-}{\lambda }(\varepsilon )(xy)\leq \max(\overset{-}{\lambda }(\varepsilon )(x),\overset{-}{\lambda }(\varepsilon )(y))$, for all ε∈A and x,y∈S.

Definition 3 Let $\lambda_{A}$ be an FBS set over *S* satisfying the condition $x\leq y\Rightarrow \overset{+}{\lambda }(\varepsilon )(x)\geq \overset{+}{\lambda }(\varepsilon )(y)$, $\overset{-}{\lambda }(\varepsilon )(x)\leq \overset{-}{\lambda }(\varepsilon )(y)$ for all ε∈A, where x,y are any elements in *S*. Then, it is called an FBS left (resp., right) ideal over *S* if and only if $\overset{+}{\lambda }(\varepsilon )(xy)\geq \overset{+}{\lambda }(\varepsilon )(y)$, $\overset{-}{\lambda }(\varepsilon )(xy)\leq \overset{-}{\lambda }(\varepsilon )(y)$ (resp., $\overset{+}{\lambda }(\varepsilon )(xy)\geq \overset{+}{\lambda }(\varepsilon )(x)$, $\overset{-}{\lambda }(\varepsilon )(xy)\leq \overset{-}{\lambda }(\varepsilon )(x)$), for all ε∈A and x,y∈S.

Moreover, $\lambda_{A}$ is called an FBS two-sided ideal or, simply, an FBS ideal over *S* if it is both an FBS left ideal and an FBS right ideal over *S*. Equivalently, we say that $\lambda_{A}$ is an FBS ideal over *S* if and only if $\overset{+}{\lambda }(\varepsilon )(xy)\geq$ $\max(\overset{+}{\lambda }(\varepsilon )(x),\overset{+}{\lambda }(\varepsilon )(y))$ and $\overset{-}{\lambda }(\varepsilon )(xy)\leq \min(\overset{-}{\lambda }(\varepsilon )(x),\overset{-}{\lambda }(\varepsilon )(y))$, for all ε∈A and x,y∈S.

Definition 4 Let $\lambda_{A}$ be an FBS ordered semigroup over *S*. Then, it is called an FBS bi-ideal over *S* if and only if $\overset{+}{\lambda }(\varepsilon )(xyz)\geq \min(\overset{+}{\lambda }(\varepsilon )(x),\overset{+}{\lambda }(\varepsilon )(z))$ and $\overset{-}{\lambda }(\varepsilon )(xyz)\leq \max(\overset{-}{\lambda }(\varepsilon )(x),\overset{-}{\lambda }(\varepsilon )(z))$, for all ε∈A and x,y∈S.

Definition 5 Let $\lambda_{A}$ be an FBS set over *S*. Then, it is called FBS prime if and only if $\overset{+}{\lambda }(\varepsilon )(xy)=\max\{\overset{+}{\lambda }(\varepsilon )(x),\overset{+}{\lambda }(\varepsilon )(y)\}$ and $\overset{-}{\lambda }(\varepsilon )(xy)=\min\{\overset{-}{\lambda }(\varepsilon )(x),\overset{-}{\lambda }(\varepsilon )(y)\}$, for all ε∈A and x,y∈S.

Definition 6 An FBS ideal $\lambda_{A}$ over *S* is called FBS prime ideal if and only if it is FBS prime. Similarly, an FBS left (resp., right) ideal $\lambda_{A}$ over *S* is called FBS prime left (resp., right) ideal if and only if it is FBS prime.

Definition 7 Let $\lambda_{A}$ be an FBS set over *S* and x∈S. Then the FBS set $<x,\lambda_{A}>$ over *S*, where

$$<x,\overset{+}{\lambda }>(\varepsilon )(y)=\overset{+}{\lambda }(\varepsilon )(xy),\;\;\;\;\;<x,\overset{-}{\lambda }>(\varepsilon )(y)=\overset{-}{\lambda }(\varepsilon )(xy),$$

for all ε∈A and y∈S, is called the extension of $\lambda_{A}$ by *x*.

Definition 8 Let *P* be a non-empty subset of *S*. Then an FBS set of the form

$$\overset{P}{\chi_{{}_{A}}}=(\overset{+}{\chi_{{}_{P}}},\overset{-}{\chi_{{}_{P}}},A)$$

over *S* is called FBS characteristic function of *P*, where

$$\overset{+}{\chi_{{}_{P}}}(\varepsilon )(x)=\left\{ \begin{array}{cc} 1, & \text{if }x\in P,\\ 0, & \text{if }x\notin P, \end{array}\right.$$

and

$$\overset{-}{\chi_{{}_{P}}}(\varepsilon )(x)=\left\{ \begin{array}{cc} 0, & \text{if }x\in P,\\ 1, & \text{if }x\notin P, \end{array}\right.$$

for all ε∈A and x∈S.

## 2 Fuzzy bipolar soft semiprimality in ordered semigroups

In this section, we introduce the concept of FBS semiprimality in ordered semigroup theory and some properties of the notion are studied. The concept is elaborated by an example. Moreover, a semiprime ideal *P* of *S* is characterized by its FBS characteristic function. If *S* is Archimedean, then it is proved that every FBS semiprime ideal $\lambda_{A}$ over *S* is a constant mapping.

Definition 9 An FBS set $\lambda_{A}$ over *S* is called FBS semiprime if and only if, for all ε∈A and x∈S, we have

$$\overset{+}{\lambda }(\varepsilon )(x)\geq \overset{+}{\lambda }(\varepsilon )(x^{2}),\;\;\;\;\;\overset{-}{\lambda }(\varepsilon )(x)\leq \overset{-}{\lambda }(\varepsilon )(x^{2}).$$

Definition 10 An FBS ideal $\lambda_{A}$ over *S* is called FBS semiprime ideal if and only if it is FBS semiprime. Similarly, an FBS left (resp., right) ideal $\lambda_{A}$ over *S* is called FBS semiprime left (resp., right) ideal if and only if it is FBS semiprime.

Example 1 Consider the ordered semigroup $S=\{\tau_{0},\tau_{1},\tau_{2},\tau_{3},\tau_{4}\}$ with the multiplication “⋅” and the order relation “≤” given below:

**Table:** 

⋅	*τ*_0_	*τ*_1_	*τ*_2_	*τ*_3_	*τ*_4_
*τ*_0_	*τ*_0_	*τ*_0_	*τ*_0_	*τ*_0_	*τ*_0_
*τ*_1_	*τ*_0_	*τ*_1_	*τ*_1_	*τ*_1_	*τ*_4_
*τ*_2_	*τ*_0_	*τ*_1_	*τ*_1_	*τ*_2_	*τ*_4_
*τ*_3_	*τ*_0_	*τ*_1_	*τ*_1_	*τ*_3_	*τ*_4_
*τ*_4_	*τ*_0_	*τ*_1_	*τ*_1_	*τ*_4_	*τ*_4_

$\leq =\{(\tau_{0},\tau_{0}),(\tau_{1},\tau_{1}),(\tau_{1},\tau_{4}),(\tau_{2},\tau_{2}),(\tau_{2},\tau_{4}),(\tau_{3},\tau_{3}),(\tau_{4},\tau_{4})\}.$

Let $E=\{\varepsilon_{1},\varepsilon_{2},\varepsilon_{3},\varepsilon_{4},\varepsilon_{5},\varepsilon_{6}\}$ be a set of parameters, $A=\{\varepsilon_{1},\varepsilon_{3},\varepsilon_{5}\}$ be its subset and f:A→E be an injective function defined by $f(\varepsilon_{i})=\varepsilon_{i+1}$ (i=1,3,5). Further, let $\lambda_{A}$ be an FBS left ideal over *S* defined as follows:

$$\overset{+}{\lambda }(\varepsilon_{1})(x)=\left\{ \begin{array}{cc} 0.6 & \text{if }x\in \{\tau_{1},\tau_{2}\},\\ 0.7 & \text{if }x=\tau_{0},\\ 0.5 & \text{if }x\in \{\tau_{3},\tau_{4}\}, \end{array}\right.$$

$$\overset{+}{\lambda }(\varepsilon_{3})(x)=\left\{ \begin{array}{cc} 0.4 & \text{if }x\in \{\tau_{1},\tau_{2}\},\\ 0.6 & \text{if }x=\tau_{0},\\ 0.2 & \text{if }x\in \{\tau_{3},\tau_{4}\}, \end{array}\right.$$

$$\overset{+}{\lambda }(\varepsilon_{5})(x)=\left\{ \begin{array}{cc} 0.3 & \text{if }x\in \{\tau_{1},\tau_{2}\},\\ 0.5 & \text{if }x=\tau_{0},\\ 0.1 & \text{if }x\in \{\tau_{3},\tau_{4}\}, \end{array}\right.$$

$$\overset{-}{\lambda }(\varepsilon_{2})(x)=\left\{ \begin{array}{cc} 0.6 & \text{if }x\in \{\tau_{1},\tau_{2}\},\\ 0.5 & \text{if }x=\tau_{0},\\ 0.7 & \text{if }x\in \{\tau_{3},\tau_{4}\}. \end{array}\right.$$

$$\overset{-}{\lambda }(\varepsilon_{4})(x)=\left\{ \begin{array}{cc} 0.4 & \text{if }x\in \{\tau_{1},\tau_{2}\},\\ 0.2 & \text{if }x=\tau_{0},\\ 0.6 & \text{if }x\in \{\tau_{3},\tau_{4}\}. \end{array}\right.$$

$$\overset{-}{\lambda }(\varepsilon_{6})(x)=\left\{ \begin{array}{cc} 0.5 & \text{if }x\in \{\tau_{1},\tau_{2}\},\\ 0.3 & \text{if }x=\tau_{0},\\ 0.6 & \text{if }x\in \{\tau_{3},\tau_{4}\}. \end{array}\right.$$

Since

$$\overset{+}{\lambda }(\varepsilon )(x)=\overset{+}{\lambda }(\varepsilon )(x^{2}),\;\;\;\;\overset{-}{\lambda }(\varepsilon )(x)=\overset{-}{\lambda }(\varepsilon )(x^{2}),$$

for all ε∈A and x∈S, thus, $\lambda_{A}$ is FBS semiprime.

Proposition 1 *Let* $\lambda_{A}$ *be an FBS ordered semigroup over S and* a∈S*. If* $\lambda_{A}$ *is FBS semiprime, then the assertions*

$$\overset{+}{\lambda }(\varepsilon )(a)=\overset{+}{\lambda }(\varepsilon )(a^{2}),\;\;\;\;\overset{-}{\lambda }(\varepsilon )(a)=\overset{-}{\lambda }(\varepsilon )(a^{2}),$$

*hold, for all* ε∈A *and* a∈S*.*

Proof Since $\lambda_{A}$ is an FBS ordered semigroup over *S*, thus we have

$$\overset{+}{\lambda }(\varepsilon )(a)\geq \overset{+}{\lambda }(\varepsilon )(a^{2})\geq \min\{\overset{+}{\lambda }(\varepsilon )(a),\overset{+}{\lambda }(\varepsilon )(a)\}=\overset{+}{\lambda }(\varepsilon )(a),$$

and

$$\overset{-}{\lambda }(\varepsilon )(a)\leq \overset{-}{\lambda }(\varepsilon )(a^{2})\leq \max\{\overset{-}{\lambda }(\varepsilon )(a),\overset{-}{\lambda }(\varepsilon )(a)\}=\overset{-}{\lambda }(\varepsilon )(a),$$

for all ε∈A and a∈S. Thus, the proof of the proposition follows. □

The following theorem explains that the concept of FBS semiprimality in ordered semigroups is, in fact, an extension of semiprimality in ordered semigroup theory.

Theorem 1 *Let P be a nonempty subset of S. Then the following conditions are equivalent.*

(1) *P is semiprime.*
(2) *The FBS characteristic function* $\overset{P}{\chi_{{}_{A}}}$ *of P is FBS semiprime.*

Proof First assume that *P* is semiprime and let $\overset{P}{\chi_{{}_{A}}}$ be the FBS characteristic function of *P*. Let ε∈A and a∈S. If $a^{2}\notin P$, then

$${\overset{+}{\chi }}_{{}_{P}}(\varepsilon )(a^{2})=0,\;\;\;\;{\overset{-}{\chi }}_{{}_{\Lambda }}(\varepsilon )(a^{2})=1.$$

Since

$${\overset{+}{\chi }}_{{}_{P}}(\varepsilon )(a)\geq 0,\;\;\;\;{\overset{-}{\chi }}_{{}_{P}}(\varepsilon )(a)\leq 1,$$

thus, we have

$${\overset{+}{\chi }}_{{}_{P}}(\varepsilon )(a)\geq {\overset{+}{\chi }}_{{}_{P}}(\varepsilon )(a^{2}),\;\;\;\;{\overset{-}{\chi }}_{{}_{P}}(\varepsilon )(a)\leq {\overset{-}{\chi }}_{{}_{P}}(\varepsilon )(a^{2}).$$

If $a^{2}\in P$, then

$${\overset{+}{\chi }}_{{}_{P}}(\varepsilon )(a^{2})=1,\;\;\;\;{\overset{-}{\chi }}_{{}_{P}}(\varepsilon )(a^{2})=0.$$

Since *P* is semiprime, thus, we have a∈P. Then, it follows that

$${\overset{+}{\chi }}_{{}_{P}}(\varepsilon )(a)=1,\;\;\;\;{\overset{-}{\chi }}_{{}_{P}}(\varepsilon )(a)=0.$$

So, in this case, we obtain

$${\overset{+}{\chi }}_{{}_{P}}(\varepsilon )(a)={\overset{+}{\chi }}_{{}_{P}}(\varepsilon )(a^{2}),\;\;\;\;{\overset{-}{\chi }}_{{}_{P}}(\varepsilon )(a)={\overset{-}{\chi }}_{{}_{P}}(\varepsilon )(a^{2}).$$

Therefore, $\overset{P}{\chi_{{}_{A}}}$ is FBS semiprime. Conversely, let the FBS characteristic function $\overset{P}{\chi_{{}_{A}}}$ of *P* is FBS semiprime. Suppose ε∈A and *a* be an element in *S* such that $a^{2}\in P$. Then, by the hypothesis, we have

$${\overset{+}{\chi }}_{{}_{P}}(\varepsilon )(a)\geq {\overset{+}{\chi }}_{{}_{P}}(\varepsilon )(a^{2}),\;\;\;\;{\overset{-}{\chi }}_{{}_{P}}(\varepsilon )(a)\leq {\overset{-}{\chi }}_{{}_{P}}(\varepsilon )(a^{2}).$$

Further, since $a^{2}\in P$, we have

$${\overset{+}{\chi }}_{{}_{P}}(\varepsilon )(a^{2})=1,\;\;\;\;{\overset{-}{\chi }}_{{}_{P}}(\varepsilon )(a^{2})=0.$$

Thus, we obtain

$${\overset{+}{\chi }}_{{}_{P}}(\varepsilon )(a)=1,\;\;\;\;{\overset{-}{\chi }}_{{}_{P}}(\varepsilon )(a)=0,$$

which implies that a∈P. Therefore, *P* is semiprime. The proof of the proposition is completed. □

Similarly, the following theorem explains that the concept of FBS primality in ordered semigroups is, in fact, an extension of primality in ordered semigroup theory.

Theorem 2 *Let P be a nonempty subset of S. Then the following conditions are equivalent.*

(1) *P is prime.*
(2) *The FBS characteristic function* $\overset{P}{\chi_{{}_{A}}}$ *of P is FBS prime.*

Proof It is straightforward. □

Proposition 2 *Let* $\lambda_{A}$ *be an FBS semiprime left (resp., right, two-sided) ideal over S and* a∈S*. Then, for all* n∈N *and for all* ε∈A*, we have*

$$\overset{+}{\lambda }(\varepsilon )(a)=\overset{+}{\lambda }(\varepsilon )(a^{n}),\;\;\;\;\overset{-}{\lambda }(\varepsilon )(a)=\overset{-}{\lambda }(\varepsilon )(a^{n}).$$

Proof Let *ε* be an arbitrary element in *A*. For n=2, we have

$$\overset{+}{\lambda }(\varepsilon )(a^{2})=\overset{+}{\lambda }(\varepsilon )(aa)\geq \overset{+}{\lambda }(\varepsilon )(a)\geq \overset{+}{\lambda }(\varepsilon )(a^{2}),$$

and

$$\overset{-}{\lambda }(\varepsilon )(a^{2})=\overset{-}{\lambda }(\varepsilon )(aa)\leq \overset{-}{\lambda }(\varepsilon )(a)\leq \overset{-}{\lambda }(\varepsilon )(a^{2}).$$

Suppose, for n≥2, we have

$$\overset{+}{\lambda }(\varepsilon )(a)=\overset{+}{\lambda }(\varepsilon )(a^{n}),\;\;\;\;\;\overset{-}{\lambda }(\varepsilon )(a)=\overset{-}{\lambda }(\varepsilon )(a^{n}).$$

Then

$$\overset{+}{\lambda }(\varepsilon )(a^{n+1})=\overset{+}{\lambda }(\varepsilon )(aa^{n})\geq \overset{+}{\lambda }(\varepsilon )(a^{n})\geq \overset{+}{\lambda }(\varepsilon )(a^{2n})=\overset{+}{\lambda }(\varepsilon )(a^{n-1}a^{n+1})\geq \overset{+}{\lambda }(\varepsilon )(a^{n+1}),$$

and

$$\overset{-}{\lambda }(\varepsilon )(a^{n+1})=\overset{-}{\lambda }(\varepsilon )(aa^{n})\leq \overset{-}{\lambda }(\varepsilon )(a^{n})\leq \overset{-}{\lambda }(\varepsilon )(a^{2n})=\overset{-}{\lambda }(\varepsilon )(a^{n-1}a^{n+1})\leq \overset{-}{\lambda }(\varepsilon )(a^{n+1}).$$

Thus, we obtain

$$\overset{+}{\lambda }(\varepsilon )(a^{n+1})=\overset{+}{\lambda }(\varepsilon )(a^{n})=\overset{+}{\lambda }(\varepsilon )(a),$$

and

$$\overset{-}{\lambda }(\varepsilon )(a^{n+1})=\overset{-}{\lambda }(\varepsilon )(a^{n})=\overset{-}{\lambda }(\varepsilon )(a).$$

Similarly, the other parts of the proposition are proved. □

Definition 11 [34] An ordered semigroup *S* is called Archimedean if, for any a,b∈S, there exists a positive integer *n* such that $b^{n}\in \left(SaS\right]$ (or $a^{n}\in \left(SbS\right])$. That is, for all a,b∈S there exists n∈N such that $b^{n}\leq xay$ for some x,y∈S.

In the following proposition, we give a characterization of Archimedean ordered semigroups by their FBS semiprime ideals.

Proposition 3 *Let S be Archimedean. Then every FBS semiprime ideal* $\lambda_{A}$ *over S is a constant mapping.*

Proof Suppose that $\lambda_{A}$ be an FBS semiprime ideal over *S*. Let a,b∈S and ε∈A. Since *S* is Archimedean, so there exist elements *x*, *y* in *S* such that $a^{n}\leq xby$ and $b^{n}\leq xay$ for some n∈N. Then, by Proposition 2, we have

$$\overset{+}{\lambda }(\varepsilon )(a)=\overset{+}{\lambda }(\varepsilon )(a^{n})\geq \overset{+}{\lambda }(\varepsilon )(xby)\geq \overset{+}{\lambda }(\varepsilon )(b),$$

and

$$\overset{-}{\lambda }(\varepsilon )(a)=\overset{-}{\lambda }(\varepsilon )(a^{n})\leq \overset{-}{\lambda }(\varepsilon )(xby)\leq \overset{-}{\lambda }(\varepsilon )(b).$$

Similarly, we have

$$\overset{+}{\lambda }(\varepsilon )(b)=\overset{+}{\lambda }(\varepsilon )(b^{n})\geq \overset{+}{\lambda }(\varepsilon )(xay)\geq \overset{+}{\lambda }(\varepsilon )(a),$$

and

$$\overset{-}{\lambda }(\varepsilon )(b)=\overset{-}{\lambda }(\varepsilon )(b^{n})\leq \overset{-}{\lambda }(\varepsilon )(xay)\leq \overset{-}{\lambda }(\varepsilon )(a).$$

Thus, we obtain

$$\overset{+}{\lambda }(\varepsilon )(a)=\overset{+}{\lambda }(\varepsilon )(b),\;\;\;\;\overset{-}{\lambda }(\varepsilon )(a)=\overset{-}{\lambda }(\varepsilon )(b).$$

Therefore, $\lambda_{A}$ is constant. □

## 3 Characterizations of left (resp., right) regular ordered semigroups by fuzzy bipolar soft semiprimality

In this section, we characterize left regular (resp., right regular) ordered semigroups in terms of FBS semiprimality of their left (resp., right) ideals. An ordered semigroup *S* is called left regular (resp., right regular) if, for every x∈S, there exists a∈S such that $x\leq ax^{2}$ (resp., $x\leq x^{2}a$). Similarly, *S* is called regular if, for any a∈S, there exists x∈S such that a≤axa i.e., a∈(aSa] for every a∈S or A⊆(ASA] for every A⊆S [35], [36].

Lemma 1 *Let P be a nonempty subset of S. Then the following conditions are equivalent on S:*

(1) *P is a left (resp., right, two-sided) ideal of S.*
(2) *The FBS characteristic function* $\overset{P}{\chi_{{}_{A}}}$ *of P is an FBS left (resp., right, two-sided) ideal over S.*

Proof First assume that Condition (1) holds, and $\overset{P}{\chi_{{}_{A}}}$ be an FBS characteristic function of *P*. Let ε∈A and μ,ν∈S. If ν∈P, then μν∈P for all μ∈S. Thus, we have

$${\overset{+}{\chi }}_{{}_{P}}(\varepsilon )(\mu \nu )=1,\;\;\;\;\;{\overset{-}{\chi }}_{{}_{P}}(\varepsilon )(\mu \nu )=0.$$

So, it follows that

$${\overset{+}{\chi }}_{{}_{P}}(\varepsilon )(\mu \nu )\geq {\overset{+}{\chi }}_{{}_{P}}(\varepsilon )(\nu ),\;\;\;\;{\overset{-}{\chi }}_{{}_{P}}(\varepsilon )(\mu \nu )\leq {\overset{-}{\chi }}_{{}_{P}}(\varepsilon )(\nu ).$$

Let ν∉P, then

$${\overset{+}{\chi }}_{{}_{P}}(\varepsilon )(\nu )=0,\;\;\;{\overset{-}{\chi }}_{{}_{P}}(\varepsilon )(\nu )=1,$$

which implies that

$${\overset{+}{\chi }}_{{}_{P}}(\varepsilon )(\mu \nu )\geq {\overset{+}{\chi }}_{{}_{P}}(\varepsilon )(\nu ),\;\;\;\;{\overset{-}{\chi }}_{{}_{P}}(\varepsilon )(\mu \nu )\leq {\overset{-}{\chi }}_{{}_{P}}(\varepsilon )(\nu ).$$

Now, let ν∈P such that S∋μ≤ν. Then

$${\overset{+}{\chi }}_{{}_{P}}(\varepsilon )(\nu )=1,\;\;\;\;{\overset{-}{\chi }}_{{}_{P}}(\varepsilon )(\nu )=0,$$

which implies that

$${\overset{+}{\chi }}_{{}_{P}}(\varepsilon )(\nu )\geq {\overset{+}{\chi }}_{{}_{P}}(\varepsilon )(\mu ),\;\;\;\;\;{\overset{-}{\chi }}_{{}_{P}}(\varepsilon )(\nu )\leq {\overset{-}{\chi }}_{{}_{P}}(\varepsilon )(\mu ).$$

Consequently, $\overset{P}{\chi_{{}_{A}}}$ is an FBS left ideal over *S*. Conversely, assume that Condition (2) holds. Let ε∈A, and let μ,ν∈S such that ν∈P. Then

$${\overset{+}{\chi }}_{{}_{P}}(\varepsilon )(\nu )=1,\;\;\;\;\;{\overset{-}{\chi }}_{{}_{P}}(\varepsilon )(\nu )=0.$$

Further, by the hypothesis, we have

$${\overset{+}{\chi }}_{{}_{P}}(\varepsilon )(\mu \nu )\geq {\overset{+}{\chi }}_{{}_{P}}(\varepsilon )(\nu ),\;\;\;\;{\overset{-}{\chi }}_{{}_{P}}(\varepsilon )(\mu \nu )\leq {\overset{-}{\chi }}_{{}_{P}}(\varepsilon )(\nu ).$$

Thus, it follows that

$${\overset{+}{\chi }}_{{}_{P}}(\varepsilon )(\mu \nu )=1,\;\;\;\;\;{\overset{-}{\chi }}_{{}_{P}}(\varepsilon )(\mu \nu )=0,$$

which implies that μν∈P. Now, let σ,τ∈S such that σ≤τ. Suppose τ∈P. By the hypothesis ${\overset{P}{\chi }}_{{}_{A}}$ is an FBS left ideal over *S*, thus, we have

$${\overset{+}{\chi }}_{{}_{P}}(\varepsilon )(\sigma )\geq {\overset{+}{\chi }}_{{}_{P}}(\varepsilon )(\tau )=1,\;\;\;\;\;{\overset{-}{\chi }}_{{}_{P}}(\varepsilon )(\sigma )\leq {\overset{-}{\chi }}_{{}_{P}}(\varepsilon )(\tau )=0.$$

Thus, it follows that

$${\overset{+}{\chi }}_{{}_{P}}(\varepsilon )(\sigma )=1,\;\;\;\;\;{\overset{-}{\chi }}_{{}_{P}}(\varepsilon )(\sigma )=0,$$

which implies that σ∈P. Therefore, *P* is a left ideal of *S*. In the same way, the other parts of the lemma are proved. □

In the following, we characterize left regular ordered semigroups by the FBS semiprimality of their FBS left ideals.

Proposition 4 *The following assertions are equivalent on S.*

(1) *S is left regular.*
(2) *Every FBS left ideal over S is FBS semiprime.*

Proof Let's first assume that Assertion (1) holds. Suppose that $\lambda_{A}$ be an FBS left ideal over *S* and σ∈S. Then, $\sigma \leq x\sigma^{2}$ for some x∈S because *S* is left regular. So, for all ε∈A, we have

$$\overset{+}{\lambda }(\varepsilon )(\sigma )\geq \overset{+}{\lambda }(\varepsilon )(x\sigma^{2})\geq \overset{+}{\lambda }(\varepsilon )(\sigma^{2}),$$

and

$$\overset{-}{\lambda }(\varepsilon )(\sigma )\leq \overset{-}{\lambda }(\varepsilon )(x\sigma^{2})\leq \overset{-}{\lambda }(\varepsilon )(\sigma^{2}).$$

Thus $\lambda_{A}$ is semiprime and that Assertion (2) holds. Conversely, assume that Assertion (2) holds and let σ∈S. Since $L(\sigma^{2})=\left(\sigma^{2}\cup S\sigma^{2}\right]$ is a left ideal of *S* generated by $\sigma^{2}$, thus, by Lemma 1, the FBS characteristic function $\overset{L(\sigma^{2})}{\chi_{{}_{A}}}$ of $L(\sigma^{2})$ is an FBS left ideal over *S*. Then, by the hypothesis, $\overset{L(\sigma^{2})}{\chi_{{}_{A}}}$ is FBS semiprime. Thus $L(\sigma^{2})$ is, by Theorem 1, semiprime. Further, since $\sigma^{2}\in L(\sigma^{2})$, we have $a\in L(\sigma^{2})=\left(\sigma^{2}\cup S\sigma^{2}\right]$. This implies that $a\leq \sigma^{2}$ or $a\leq x\sigma^{2}$ for some x∈S. If $\sigma \leq \sigma^{2}$, then $\sigma \leq \sigma^{2}=\sigma \sigma \leq \sigma \sigma^{2}\in S\sigma^{2}$ which implies that $\sigma \in \left(S\sigma^{2}\right]$. Similarly, if $\sigma \leq x\sigma^{2}$, we obtain $\sigma \in \left(S\sigma^{2}\right]$. Thus, *S* is left regular, and that Assertion (1) holds. □

As an application of Proposition 4, we present the following example.

Example 2 Consider the ordered semigroup $S=\{\tau_{0},\tau_{1},\tau_{2},\tau_{3}\}$ with the multiplication “⋅” and the order relation “≤” given below:

**Table:** 

⋅	*τ*_0_	*τ*_1_	*τ*_2_	*τ*_3_
*τ*_0_	*τ*_2_	*τ*_2_	*τ*_2_	*τ*_2_
*τ*_1_	*τ*_2_	*τ*_2_	*τ*_2_	*τ*_2_
*τ*_2_	*τ*_2_	*τ*_2_	*τ*_2_	*τ*_2_
*τ*_3_	*τ*_0_	*τ*_1_	*τ*_2_	*τ*_3_

$\leq =\{(\tau_{0},\tau_{0}),(\tau_{0},\tau_{2}),(\tau_{1},\tau_{1}),(\tau_{1},\tau_{2}),(\tau_{2},\tau_{2}),(\tau_{3},\tau_{3})\}.$

One can check that (S,⋅,≤) is a left regular ordered semigroup.

Now, let $E=\{\varepsilon_{1},\varepsilon_{2},\varepsilon_{3},\varepsilon_{4},\varepsilon_{5}\}$ be a set of parameters and $A=\{\varepsilon_{1},\varepsilon_{2},\varepsilon_{3}\}$ be its subset. Further, let f:A→E be an injective function such that $f(\varepsilon_{1})=\varepsilon_{1}$, $f(\varepsilon_{2})=\varepsilon_{4}$, $f(\varepsilon_{3})=\varepsilon_{5}$.

Let's define an FBS left ideal $\Gamma_{A}$ over *S* as follows:

$$\overset{+}{\Gamma }(\varepsilon_{1})(x)=\left\{ \begin{array}{cc} 0.6 & \text{if }x\in \{\tau_{0},\tau_{1},\tau_{2}\},\\ 0.4 & \text{if }x=\tau_{3}, \end{array}\right.$$

$$\overset{+}{\Gamma }(\varepsilon_{2})(x)=\left\{ \begin{array}{cc} 0.5 & \text{if }x\in \{\tau_{0},\tau_{1},\tau_{2}\},\\ 0.3 & \text{if }x=\tau_{3}, \end{array}\right.$$

$$\overset{+}{\Gamma }(\varepsilon_{3})(x)=\left\{ \begin{array}{cc} 0.4 & \text{if }x\in \{\tau_{0},\tau_{1},\tau_{2}\},\\ 0.2 & \text{if }x=\tau_{3}, \end{array}\right.$$

$$\overset{-}{\Gamma }(\varepsilon_{1})(x)=\left\{ \begin{array}{cc} 0.3 & \text{if }x\in \{\tau_{0},\tau_{1},\tau_{2}\},\\ 0.5 & \text{if }x=\tau_{3}, \end{array}\right.$$

$$\overset{-}{\Gamma }(\varepsilon_{4})(x)=\left\{ \begin{array}{cc} 0.3 & \text{if }x\in \{\tau_{0},\tau_{1},\tau_{2}\},\\ 0.5 & \text{if }x=\tau_{3}, \end{array}\right.$$

$$\overset{-}{\Gamma }(\varepsilon_{5})(x)=\left\{ \begin{array}{cc} 0.4 & \text{if }x\in \{\tau_{0},\tau_{1},\tau_{2}\},\\ 0.5 & \text{if }x=\tau_{3}. \end{array}\right.$$

Then, by virtue of Proposition 4, we have $\Gamma_{A}$ is FBS semiprime. Independently, one can check that $\Gamma_{A}$ is FBS semiprime.

To prove our next theorem, we need the following result:

Lemma 2 *[36]* *Let* σ∈S*. Then* $\left(S\sigma^{2}\right]$ *(resp.,* $\left(\sigma^{2}S\right]$ *is a left (resp., right) ideal of S.*

Theorem 3 *The following assertions are equivalent on S.*

(1) *S is left regular.*
(2) *Every left ideal of S is semiprime.*
(3) *Every FBS left ideal* $\lambda_{A}$ *over S is FBS semiprime.*
(4) *If* $\lambda_{A}$ *is an FBS left ideal over S, then, for all* ε∈A *and* a∈S*,*
  $$\overset{+}{\lambda }(a)=\overset{+}{\lambda }(a^{2}),\;\;\;\;\overset{-}{\lambda }(a)=\overset{-}{\lambda }(a^{2}).$$
(5) $a\in L(a^{2})$ *for every* a∈S*.*
(6) $L(a)=L(a^{2})$ *for every* a∈S*.*

Proof First assume (1) holds. In order to prove that Assertion (2) holds, let *L* be a left ideal of *S*. Suppose $a^{2}\in L$, for some a∈S. Then, since *S* is left regular, we have $a\in \left(Sa^{2}\right]\subseteq \left(SL\right]\subseteq \left(L\right]=L$. Thus *L* is semiprime and that Assertion (1) implies (2). Now, assume that (2) holds. Let $\lambda_{A}$ be an FBS left ideal over *S* and a∈S. By Lemma 2, the set $\left(Sa^{2}\right]$ is a left ideal of *S*. Then, by the hypothesis, $\left(Sa^{2}\right]$ is semiprime. Since $a^{4}\in \left(Sa^{2}\right]$, we have

$$a^{2}\in \left(Sa^{2}\right]\Rightarrow \;a\in \left(Sa^{2}\right].$$

Then $a\leq xa^{2}$ for some x∈S. Thus, for all ε∈A, we have

$$\overset{+}{\lambda }(\varepsilon )(a)\geq \overset{+}{\lambda }(\varepsilon )(xa^{2})\geq \overset{+}{\lambda }(\varepsilon )(a^{2}),$$

and

$$\overset{-}{\lambda }(\varepsilon )(a)\leq \overset{-}{\lambda }(\varepsilon )(xa^{2})\leq \overset{-}{\lambda }(\varepsilon )(a^{2}).$$

Therefore, $\lambda_{A}$ is FBS semiprime and that Assertion (2) implies (3). Next, assume that (3) holds. Let $\lambda_{A}$ be a FBS left ideal over *S*. Let a∈S and ε∈A. Since, by the hypothesis, $\lambda_{A}$ is FBS semiprime, then

$$\overset{+}{\lambda }(\varepsilon )(a)\geq \overset{+}{\lambda }(\varepsilon )(a^{2})\geq \overset{+}{\lambda }(\varepsilon )(a),$$

and

$$\overset{-}{\lambda }(\varepsilon )(a)\leq \overset{-}{\lambda }(\varepsilon )(a^{2})\leq \overset{-}{\lambda }(\varepsilon )(a).$$

Therefore, we obtain

$$\overset{+}{\lambda }(\varepsilon )(a)=\overset{+}{\lambda }(\varepsilon )(a^{2}),\;\;\;\;\overset{-}{\lambda }(\varepsilon )(a)=\overset{-}{\lambda }(\varepsilon )(a^{2}).$$

Thus Assertion (3) implies (4). Now, assume that (4) holds. Let a∈S and ε∈A. Since $L(a^{2})$ is a left ideal of *S*, thus, by Lemma 1, the FBS characteristic function $\overset{L(a^{2})}{\chi_{{}_{A}}}$ of $L(a^{2})$ is an FBS left ideal over *S*. Then, by the hypothesis, we have

$${\overset{+}{\chi }}_{{}_{L(a^{2})}}(\varepsilon )(a)={\overset{+}{\chi }}_{{}_{L(a^{2})}}(\varepsilon )(a^{2})=1,$$

$${\overset{-}{\chi }}_{{}_{L(a^{2})}}(\varepsilon )(a)={\overset{-}{\chi }}_{{}_{L(a^{2})}}(\varepsilon )(a^{2})=0.$$

This implies that $a\in L(a^{2})$ and that Assertion (4) implies (5). Now, assume that Assertion (5) holds. Let a∈S. Then, we have

$$a\in L(a^{2})=\left(a^{2}\cup Sa^{2}\right]\subseteq \left(Sa\right]\subseteq L(a)\subseteq L(a^{2}).$$

Thus, we obtain $L(a)=L(a^{2})$ and that (5) implies (6). Finally, assume that (6) holds. Let a∈S. Then, we have

$$a\in L(a)=L(a^{2})=\left(a^{2}\cup Sa^{2}\right].$$

Further,

$$a^{2}\in \left(a^{2}\cup Sa^{2}\right]\left(a\right]\subseteq \left(a^{3}\cup Sa^{3}\right]\subseteq \left(Sa^{2}\right].$$

Thus, we have

$$a\in \left(\left(Sa^{2}\right]\cup Sa^{2}\right]\subseteq \left(\left(Sa^{2}\right]\right]=\left(Sa^{2}\right].$$

Thus, *S* is left regular and that Assertion (6) implies (1). The proof of the theorem is completed. □

In a similar fashion, in the following, right regular ordered semigroups are characterized by FBS semiprimality of their FBS right ideals.

Proposition 5 *The following assertions are equivalent on S.*

(1) *S is right regular.*
(2) *Every FBS right ideal over S is FBS semiprime.*

Proof It is straightforward. □

As an application of Proposition 5, we display the following example.

Example 3 Consider the ordered semigroup $S=\{\tau_{0},\tau_{1},\tau_{2},\tau_{3}\}$ with the multiplication “⋅” and the order relation “≤” given below:

**Table:** 

⋅	*τ*_0_	*τ*_1_	*τ*_2_	*τ*_3_
*τ*_0_	*τ*_0_	*τ*_0_	*τ*_0_	*τ*_0_
*τ*_1_	*τ*_0_	*τ*_1_	*τ*_1_	*τ*_3_
*τ*_2_	*τ*_0_	*τ*_1_	*τ*_1_	*τ*_3_
*τ*_3_	*τ*_0_	*τ*_1_	*τ*_1_	*τ*_3_

$\leq =\{(\tau_{0},\tau_{0}),(\tau_{1},\tau_{1}),(\tau_{1},\tau_{3}),(\tau_{2},\tau_{2}),(\tau_{2},\tau_{3}),(\tau_{3},\tau_{3})\}.$

One can check that (S,⋅,≤) is a right regular ordered semigroup.

Now, let $E=Z_{4}=\{0,1,2,3\}$ be a set of parameters, A={0,2} be its subset and f:A→E be an injective function given by $f(\varepsilon )=\varepsilon^{-1}$, for all ε∈A. Let's define an FBS right ideal $\Gamma_{A}$ over *S* as follows:

$$\overset{+}{\Gamma }(0)(x)=\left\{ \begin{array}{cc} 0.4 & \text{if }x\in \{\tau_{1},\tau_{2},\tau_{3}\},\\ 0.6 & \text{if }x=\tau_{0}, \end{array}\right.$$

$$\overset{+}{\Gamma }(2)(x)=\left\{ \begin{array}{cc} 0.2 & \text{if }x\in \{\tau_{1},\tau_{2},\tau_{3}\},\\ 0.5 & \text{if }x=\tau_{0}, \end{array}\right.$$

$$\overset{-}{\Gamma }(0)(x)=\left\{ \begin{array}{cc} 0.5 & \text{if }x\in \{\tau_{1},\tau_{2},\tau_{3}\},\\ 0.3 & \text{if }x=\tau_{0}, \end{array}\right.$$

$$\overset{-}{\Gamma }(2)(x)=\left\{ \begin{array}{cc} 0.6 & \text{if }x\in \{\tau_{1},\tau_{2},\tau_{3}\},\\ 0.3 & \text{if }x=\tau_{0}. \end{array}\right.$$

Then, by Proposition 5, we have $\Gamma_{A}$ is FBS semiprime. One can check independently that $\Gamma_{A}$ is FBS semiprime.

The left-right dual of Theorem 3 holds which is formulated as follows:

Theorem 4 *The following assertions are equivalent on S.*

(1) *S is right regular.*
(2) *Every right ideal of S is semiprime.*
(3) *Every FBS right* $\lambda_{A}$ *over S is FBS semiprime.*
(4) *If* $\lambda_{A}$ *is a FBS right ideal over S, then, for all* ε∈A *and* σ∈S*,*
  $$\overset{+}{\lambda }(\sigma )=\overset{+}{\lambda }(\sigma^{2}),\;\;\;\;\overset{-}{\lambda }(\sigma )=\overset{-}{\lambda }(\sigma^{2}).$$
(5) $\sigma \in R(\sigma^{2})$ *for every* σ∈S*.*
(6) $R(\sigma )=R(\sigma^{2})$ *for every* σ∈S*.*

## 4 Characterization of completely (resp., intra-regular) ordered semigroups by fuzzy bipolar soft semiprimality

In this section, we characterize completely regular (resp., intra-regular) ordered semigroups in terms of FBS semiprime (left, right) ideals. An ordered semigroup *S* is called completely regular if it is at the same time left regular, right regular and regular. Similarly, *S* is called intra-regular if for every x∈S there exist a,b∈S such that $x\leq ax^{2}b$ i.e., $x\in \left(Sx^{2}S\right]$ or $A\subseteq \left(SA^{2}S\right]$ for all A⊆S [36], [37], [38].

Lemma 3 *[36]* *An ordered semigroup S is completely regular if and only if, for every* a∈S*, we have* $a\in \left(a^{2}Sa^{2}\right]$*.*

In the following proposition, completely regular ordered semigroups are characterized by FBS semiprimality of their left (resp., right) ideals.

Proposition 6 *Let* $\lambda_{A}$ *be an FBS left (resp., right) ideal over S. If S is completely regular, then* $\lambda_{A}$ *is FBS semiprime.*

Proof Let $\lambda_{A}$ be an FBS left ideal over *S* and σ∈S. Then, since *S* is completely regular, there exists an element *x* in *S* such that $\sigma \leq \sigma^{2}x\sigma^{2}$. So, for all ε∈A, we have

$$\overset{+}{\lambda }(\varepsilon )(\sigma )\geq \overset{+}{\lambda }(\varepsilon )(\sigma^{2}x\sigma^{2})\geq \overset{+}{\lambda }(\varepsilon )(x\sigma^{2})\geq \overset{+}{\lambda }(\varepsilon )(\sigma^{2}),$$

and

$$\overset{-}{\lambda }(\varepsilon )(\sigma )\leq \overset{-}{\lambda }(\varepsilon )(\sigma^{2}x\sigma^{2})\leq \overset{-}{\lambda }(\varepsilon )(x\sigma^{2})\leq \overset{-}{\lambda }(\varepsilon )(\sigma^{2}).$$

Thus $\lambda_{A}$ is FBS semiprime. □

The combined effect of Proposition 6 is formulated as follows:

Proposition 7 *Let* $\lambda_{A}$ *be an FBS ideal over S. If S is completely regular, then* $\lambda_{A}$ *is FBS semiprime.*

Similarly, the following proposition is established.

Proposition 8 *Let* $\lambda_{A}$ *be an FBS left (resp., right) ideal over S. If S is completely regular, then* $<x,\lambda_{A}>$ *is FBS semiprime.*

The combined effect of Proposition 8 is formulated as follows:

Proposition 9 *Let* $\lambda_{A}$ *be an FBS ideal over S. If S is completely regular, then* $<x,\lambda_{A}>$ *is FBS semiprime, for all* x∈S*.*

As an explanation of Proposition 9, we incorporate the following example.

Example 4 Consider the ordered semigroup S={0,a,b,c,d} with the multiplication “⋅” and the order relation “≤” given below:

**Table:** 

⋅	0	*a*	*b*	*c*	*d*
0	0	0	0	0	0
*a*	0	*a*	*b*	*b*	*d*
*b*	0	*b*	*b*	*b*	*b*
*c*	0	*b*	*b*	*b*	*b*
*d*	0	*d*	*b*	*b*	*d*

≤={(0,0),(a,a),(a,d),(b,b),(c,c),(c,b),(d,d)}

One can check, by routine calculations, that *S* is completely regular ordered semigroup.

Suppose $A=E=\{\varepsilon_{1},\varepsilon_{2},\varepsilon_{3}\}$ be a set of parameters and f:A→A be an identity function. Let $\lambda_{A}$ be an FBS set over *S* that is defined, for all x∈S, as follows:

$$\overset{+}{\lambda }(\varepsilon_{1})(x)=\left\{ \begin{array}{cc} 0.5 & \text{if }x\in \{0,b,c\},\\ 0.3 & \text{if }x\in \{a,d\}, \end{array}\right.$$

$$\overset{+}{\lambda }(\varepsilon_{2})(x)=\left\{ \begin{array}{cc} 0.6 & \text{if }x\in \{0,b,c\},\\ 0.4 & \text{if }x\in \{a,d\}, \end{array}\right.$$

$$\overset{+}{\lambda }(\varepsilon_{3})(x)=\left\{ \begin{array}{cc} 0.4 & \text{if }x\in \{0,b,c\},\\ 0.3 & \text{if }x\in \{a,d\}, \end{array}\right.$$

$$\overset{-}{\lambda }(\varepsilon_{1})(x)=\left\{ \begin{array}{cc} 0.4 & \text{if }x\in \{0,b,c\},\\ 0.6 & \text{if }x\in \{a,d\}, \end{array}\right.$$

$$\overset{-}{\lambda }(\varepsilon_{2})(x)=\left\{ \begin{array}{cc} 0.3 & \text{if }x\in \{0,b,c\},\\ 0.5 & \text{if }x\in \{a,d\}, \end{array}\right.$$

$$\overset{-}{\lambda }(\varepsilon_{3})(x)=\left\{ \begin{array}{cc} 0.4 & \text{if }x\in \{0,b,c\},\\ 0.5 & \text{if }x\in \{a,d\}. \end{array}\right.$$

Then $\lambda_{A}$ is an FBS ideal over *S* which is, by Proposition 7, FBS semiprime. Next, we define the FBS extension $<y,\lambda_{A}>$ of $\lambda_{A}$, for all y∈S. For this, let *y* be any element in {0,b,c}. Then, $<y,\lambda_{A}>$ is defined, for all x∈S, as follows:

$$<y,\overset{+}{\lambda }>(\varepsilon_{1})(x)=0.5,\;\;\;\;<y,\overset{-}{\lambda }>(\varepsilon_{1})(x)=0.4,$$

$$<y,\overset{+}{\lambda }>(\varepsilon_{2})(x)=0.6,\;\;\;\;<y,\overset{-}{\lambda }>(\varepsilon_{2})(x)=0.3,$$

$$<y,\overset{+}{\lambda }>(\varepsilon_{3})(x)=0.4,\;\;\;\;<y,\overset{-}{\lambda }>(\varepsilon_{3})(x)=0.4.$$

Then, obviously, $<y,\lambda_{A}>$ is FBS semiprime. Next, let *y* be any element in {a,d}. Then $<y,\lambda_{A}>$ coincides with $\lambda_{A}$. Since $\lambda_{A}$ is FBS semiprime, then so is $<y,\lambda_{A}>$. Thus, for any y∈S, we find that $<y,\lambda_{A}>$ is FBS semiprime. Therefore, we conclude that Proposition 9 stands valid.

The following characterization of an intra-regular ordered semigroup, in terms of semiprimality and fuzzy semiprimality, is due to Theorems 23 and 24 of N. Kehayopulu [39].

Lemma 4 *Let S be an ordered semigroup. Then*

(1) *S is intra-regular if and only if every ideal of S is semiprime.*
(2) *S is intra-regular if and only if every fuzzy ideal of S is fuzzy semiprime.*

In connection with Lemma 4, we note that its Part (2) is the fuzzy analogue of its Part (1). Now, in the following, we will give a characterization of intra-regular ordered semigroups by FBS semiprimality.

Theorem 5 *The following conditions are equivalent on S:*

(1) *S is intra-regular.*
(2) *Every FBS ideal* $\lambda_{A}$ *over S is FBS semiprime.*

Proof First assume that *S* is intra-regular. Let $\lambda_{A}$ be an FBS ideal over *S* and *σ* any element in *S*. Then, $\sigma \leq x\sigma^{2}y$ for some x,y∈S because *S* is intra-regular. Thus, for all ε∈A, we have

$$\overset{+}{\lambda }(\varepsilon )(\sigma )\geq \overset{+}{\lambda }(\varepsilon )(x\sigma^{2}y)\geq \overset{+}{\lambda }(\varepsilon )(\sigma^{2}y)\geq \overset{+}{\lambda }(\varepsilon )(\sigma^{2}),$$

and

$$\overset{-}{\lambda }(\varepsilon )(\sigma )\leq \overset{-}{\lambda }(\varepsilon )(x\sigma^{2}y)\leq \overset{-}{\lambda }(\varepsilon )(\sigma^{2}y)\leq \overset{-}{\lambda }(\varepsilon )(\sigma^{2}).$$

Thus Condition (1) implies (2). Conversely, assume that $\lambda_{A}$ is an FBS ideal over *S*. We consider the ideal $I(\sigma^{2})$ of *S* generated by $\sigma^{2}$ i.e., the set

$$I(\sigma^{2})=\left(\sigma^{2}\cup S\sigma^{2}\cup \sigma^{2}S\cup S\sigma^{2}S\right].$$

By Lemma 1, the FBS characteristic function $\overset{I(\sigma^{2})}{\chi_{{}_{A}}}$ of $I(\sigma^{2})$ is an FBS ideal over *S*. Then, by the assumption, $\overset{I(\sigma^{2})}{\chi_{{}_{A}}}$ is FBS semiprime. So, for all ε∈A, we have

$${\overset{+}{\chi }}_{{}_{I(\sigma^{2})}}(\varepsilon )(\sigma )\geq {\overset{+}{\chi }}_{{}_{I(\sigma^{2})}}(\varepsilon )(\sigma^{2}),$$

and

$${\overset{-}{\chi }}_{{}_{I(\sigma^{2})}}(\varepsilon )(\sigma )\leq {\overset{-}{\chi }}_{{}_{I(\sigma^{2})}}(\varepsilon )(\sigma^{2}).$$

Since $\sigma^{2}\in I(\sigma^{2})$, thus, for all ε∈A, we have

$${\overset{+}{\chi }}_{{}_{I(\sigma^{2})}}(\varepsilon )(\sigma^{2})=1,\;\;\;\;{\overset{-}{\chi }}_{{}_{I(a^{2})}}(\varepsilon )(\sigma^{2})=0.$$

Thus, we get

$${\overset{+}{\chi }}_{{}_{I(\sigma^{2})}}(\varepsilon )(\sigma )=1,\;\;\;\;{\overset{-}{\chi }}_{{}_{I(\sigma^{2})}}(\varepsilon )(\sigma )=0.$$

Consequently, $\sigma \in I(\sigma^{2})$. If $\sigma \leq \sigma^{2}$, then

$$\sigma \leq \sigma \sigma \leq \sigma^{2}\sigma^{2}=\sigma \sigma^{2}\sigma \in S\sigma^{2}S,$$

which implies that $\sigma \in \left(S\sigma^{2}S\right]$. If $\sigma \leq x\sigma^{2}$, then

$$\sigma \leq x\sigma^{2}=x\sigma \sigma \leq x\sigma^{2}\sigma \in S\sigma^{2}S,$$

which implies that $\sigma \in \left(S\sigma^{2}S\right]$. Similarly, if $\sigma \leq \sigma^{2}x$ or $\sigma \leq x\sigma^{2}y$, we obtain

$$\sigma \in \left(S\sigma^{2}S\right].$$

Therefore, *S* is intra-regular and that Condition (2) implies (1). The proof of the theorem is completed. □

As an application of Theorem 5, we present the following example.

Example 5 Consider the ordered semigroup *S* with the multiplication “⋅” and the order relation “≤” given as in Example 1:

Then one can check, by simple calculations, that *S* is intra-regular.

Let $E=\{\varepsilon_{1},\varepsilon_{2},\varepsilon_{3},\varepsilon_{4},\varepsilon_{5},\varepsilon_{6},\varepsilon_{7}\}$ be a set of parameters and $A=\{\varepsilon_{2},\varepsilon_{4},\varepsilon_{6}\}$ be its subset. Let f:A→E be an injective function such that $f(\varepsilon_{2})=\varepsilon_{3}$, $f(\varepsilon_{4})=\varepsilon_{5},f(\varepsilon_{6})=\varepsilon_{7}$. Consider an FBS set $\lambda_{A}$ over *S* which is defined as follows:

$$\overset{+}{\lambda }(\varepsilon_{2})(x)=\left\{ \begin{array}{cc} 0.5 & \text{if }x\in \{\tau_{1},\tau_{2},\tau_{4}\},\\ 0.6 & \text{if }x=\tau_{0},\\ 0.3 & \text{if }x=\tau_{3}, \end{array}\right.$$

$$\overset{+}{\lambda }(\varepsilon_{4})(x)=\left\{ \begin{array}{cc} 0.4 & \text{if }x\in \{\tau_{1},\tau_{2},\tau_{4}\},\\ 0.5 & \text{if }x=\tau_{0},\\ 0.3 & \text{if }x=\tau_{3}, \end{array}\right.$$

$$\overset{+}{\lambda }(\varepsilon_{6})(x)=\left\{ \begin{array}{cc} 0.3 & \text{if }x\in \{\tau_{1},\tau_{2},\tau_{4}\},\\ 0.4 & \text{if }x=\tau_{0},\\ 0.2 & \text{if }x=\tau_{3}, \end{array}\right.$$

$$\overset{-}{\lambda }(\varepsilon_{3})(x)=\left\{ \begin{array}{cc} 0.4 & \text{if }x\in \{\tau_{1},\tau_{2},\tau_{4}\},\\ 0.2 & \text{if }x=\tau_{0},\\ 0.5 & \text{if }x=\tau_{3}, \end{array}\right.$$

$$\overset{-}{\lambda }(\varepsilon_{5})(x)=\left\{ \begin{array}{cc} 0.3 & \text{if }x\in \{\tau_{1},\tau_{2},\tau_{4}\},\\ 0.2 & \text{if }x=\tau_{0},\\ 0.4 & \text{if }x=\tau_{3}, \end{array}\right.$$

$$\overset{-}{\lambda }(\varepsilon_{7})(x)=\left\{ \begin{array}{cc} 0.5 & \text{if }x\in \{\tau_{1},\tau_{2},\tau_{4}\},\\ 0.4 & \text{if }x=\tau_{0},\\ 0.7 & \text{if }x=\tau_{3}. \end{array}\right.$$

Then $\lambda_{A}$ is an FBS ideal over *S*. By virtue of Theorem 5, we have $\lambda_{A}$ is FBS semiprime. Independently, one can check that $\lambda_{A}$ is FBS semiprime.

To prove our next theorem, we need the following result:

Lemma 5 *[36]* *Let* a∈S*. Then* $\left(Sa^{2}S\right]$ *is a two-sided ideal of S.*

In the following, we give a characterization of intra-regular ordered semigroups by means of principal ideals, FBS ideals, and by semiprimality (resp., FBS semiprimality) of their ideals (resp., FBS ideals).

Theorem 6 *The following conditions are equivalent on S.*

1) *S is intra-regular.*
2) *If* $\lambda_{A}$ *is an FBS ideal over S, then, for all* ε∈A *and* σ∈S*,*
  $$\overset{+}{\lambda }(\varepsilon )(\sigma )=\overset{+}{\lambda }(\varepsilon )(\sigma^{2}),\;\;\;\;\overset{+}{\lambda }(\varepsilon )(\sigma )=\overset{+}{\lambda }(\varepsilon )(\sigma^{2}).$$
3) $\sigma \in I(\sigma^{2})$ *for all* σ∈S*.*
4) $I(\sigma )=I(\sigma^{2})$ *for all* σ∈S*.*
5) *Every ideal of S is semiprime.*
6) *Every FBS ideal over S is FBS semiprime.*

Proof First, assume that *S* is intra-regular. Let $\lambda_{A}$ be an FBS ideal over *S* and σ∈S. Then, $\sigma \leq x\sigma^{2}y$ for some x,y∈S because *S* is intra-regular. So, for all ε∈A, we have

$$\overset{+}{\lambda }(\varepsilon )(\sigma )\geq \overset{+}{\lambda }(\varepsilon )(x\sigma^{2}y)\geq \overset{+}{\lambda }(\varepsilon )(\sigma^{2}y)\geq \overset{+}{\lambda }(\varepsilon )(\sigma^{2})\geq \overset{+}{\lambda }(\varepsilon )(\sigma ),$$

and

$$\overset{-}{\lambda }(\varepsilon )(\sigma )\leq \overset{-}{\lambda }(\varepsilon )(x\sigma^{2}y)\leq \overset{-}{\lambda }(\varepsilon )(\sigma^{2}y)\leq \overset{-}{\lambda }(\varepsilon )(\sigma^{2})\leq \overset{-}{\lambda }(\varepsilon )(\sigma ).$$

Thus Condition (1) implies (2). Now, assume Condition (2) holds. Let σ∈S and ε∈A. The set $I(\sigma^{2})$ is an ideal of *S*. Thus, by Lemma 1, the FBS characteristic function $\overset{I(\sigma^{2})}{\chi_{{}_{A}}}$ of $I(\sigma^{2})$ is an FBS ideal over *S*. Further, by the hypothesis, we have

$${\overset{+}{\chi }}_{{}_{I(\sigma^{2})}}(\varepsilon )(\sigma )={\overset{+}{\chi }}_{{}_{I(\sigma^{2})}}(\varepsilon )(\sigma^{2})=1,$$

and

$${\overset{-}{\chi }}_{{}_{I(\sigma^{2})}}(\varepsilon )(\sigma )={\overset{-}{\chi }}_{{}_{I(\sigma^{2})}}(\varepsilon )(\sigma^{2})=0,$$

which implies that $\sigma \in I(\sigma^{2})$. Hence, Condition (2) implies (3). Now, let Condition (3) holds. Let σ∈S. Then, since $\sigma \in I(\sigma^{2})$, we have

$$I(\sigma )\subseteq I(\sigma^{2})=\left(\sigma^{2}\cup S\sigma^{2}\cup \sigma^{2}S\cup S\sigma^{2}S\right]\subseteq \left(S\sigma \cup \sigma S\cup S\sigma S\right]\subseteq I(\sigma ).$$

Thus $I(\sigma )=I(\sigma^{2})$ and that (3) implies (4). Now, assume Condition (4) holds. Let σ∈S. Suppose *P* be an ideal of *S* such that $\sigma^{2}\in P$. Then, by the hypothesis, we have

$$\sigma \in I(\sigma )=I(\sigma^{2})\subseteq P,$$

which implies that *P* is semiprime and that Condition (4) implies (5). Next, assume that Condition (5) holds. Suppose ε∈A and σ∈S. Let $\lambda_{A}$ be an FBS ideal over *S*. By Lemma 5, the set $\left(S\sigma^{2}S\right]$ is an ideal of *S* and, by the hypothesis, it is semiprime. Since $\sigma^{4}\in \left(S\sigma^{2}S\right]$, then

$$\sigma^{2}\in \left(S\sigma^{2}S\right]\;\Rightarrow \;\sigma \in \left(S\sigma^{2}S\right].$$

This means that $\sigma \leq x\sigma^{2}y$ for some x,y∈S. Further, since $\lambda_{A}$ is an FBS ideal over *S*, we have

$$\overset{+}{\lambda }(\varepsilon )(\sigma )\geq \overset{+}{\lambda }(\varepsilon )(x\sigma^{2}y)\geq \overset{+}{\lambda }(\varepsilon )(\sigma^{2}),$$

and

$$\overset{-}{\lambda }(\varepsilon )(\sigma )\geq \overset{-}{\lambda }(\varepsilon )(x\sigma^{2}y)\leq \overset{-}{\lambda }(\varepsilon )(\sigma^{2}).$$

Therefore, $\lambda_{A}$ is semiprime and that Condition (5) implies (6). Finally, let Condition (6) holds. Then *S* is, by Theorem 5, intra-regular and that Condition (1) holds. The proof of the theorem is completed. □

To elaborate Theorem 6, we display the following example:

Example 6 Consider the ordered semigroup S={a,b,c,d,f} with the multiplication “⋅” and the order relation “≤” given as follows:

**Table:** 

⋅	*a*	*b*	*c*	*d*	*f*
*a*	*b*	*a*	*a*	*a*	*a*
*b*	*a*	*b*	*b*	*b*	*b*
*c*	*a*	*b*	*b*	*b*	*b*
*d*	*a*	*b*	*b*	*d*	*d*
*f*	*a*	*b*	*c*	*d*	*f*

≤={(a,a),(b,b),(c,c),(c,b),(d,d),(f,f),(f,d)}.

Then *S* is intra-regular ordered semigroup [37].

Let $E=\{\varepsilon_{1},\varepsilon_{2},\varepsilon_{3},\varepsilon_{4},\varepsilon_{5}\}$ be a set of parameters and $A=\{\varepsilon_{1},\varepsilon_{2}\}$ such that f:A→E be an injective function given by $f(\varepsilon_{1})=\varepsilon_{3}$, $f(\varepsilon_{2})=\varepsilon_{4}$. Further, let $\lambda_{A}$ be an FBS ideal over *S* defined as follows:

$$\overset{+}{\lambda }(\varepsilon_{1})(x)=\left\{ \begin{array}{cc} 0.5 & \text{if }x\in \{a,b,c\},\\ 0.3 & \text{if }x\in \{d,f\}, \end{array}\right.$$

$$\overset{+}{\lambda }(\varepsilon_{2})(x)=\left\{ \begin{array}{cc} 0.6 & \text{if }x\in \{a,b,c\},\\ 0.4 & \text{if }x\in \{d,f\}, \end{array}\right.$$

$$\overset{-}{\lambda }(\varepsilon_{3})(x)=\left\{ \begin{array}{cc} 0.4 & \text{if }x\in \{a,b,c\},\\ 0.6 & \text{if }x\in \{d,f\}, \end{array}\right.$$

$$\overset{-}{\lambda }(\varepsilon_{4})(x)=\left\{ \begin{array}{cc} 0.3 & \text{if }x\in \{a,b,c\},\\ 0.5 & \text{if }x\in \{d,f\}. \end{array}\right.$$

Moreover, we define the following principal ideals of *S*:

$$I(a)=I(a^{2})=\{a,b,c\},$$

$$I(b)=I(b^{2})=\{a,b,c\},$$

$$I(c)=I(c^{2})=\{a,b,c\},$$

$$I(d)=I(d^{2})=S,$$

$$I(f)=I(f^{2})=S.$$

Then, we note the following:

(1) *S* is intra-regular.
(2) For all ε∈A and σ∈S, we have
  $$\overset{+}{\lambda }(\varepsilon )(\sigma )=\overset{+}{\lambda }(\varepsilon )(\sigma^{2}),\;\;\;\;\overset{+}{\lambda }(\varepsilon )(\sigma )=\overset{+}{\lambda }(\varepsilon )(\sigma^{2}).$$
(3) $\sigma \in I(\sigma^{2})$ for all σ∈S.
(4) $I(\sigma )=I(\sigma^{2})$ for all σ∈S.
(5) The ideals of *S* are {a,b,c} and *S*, which are both semiprime.
(6) Finally, $\lambda_{A}$ is FBS semiprime ideal over *S*.

The following propositions can be easily proved.

Proposition 10 *Let S be intra-regular and commutative and* $\lambda_{A}$ *be an FBS ideal over S. Then* $<x,\lambda_{A}>$ *is FBS semiprime, for all* x∈S*.*

Proposition 11 *Let* $\lambda_{A}$ *be an FBS bi-ideal over S. If S is completely regular, then* $\lambda_{A}$ *is FBS semiprime.*

Proposition 12 *Let S be completely regular and commutative and* $\lambda_{A}$ *be an FBS bi-ideal over S. Then* $<x,\lambda_{A}>$ *is FBS semiprime, for all* x∈S*.*

Lemma 6 *[36]* *The set* $\left(\sigma^{2}S\sigma^{2}\right]$ *is a bi-ideal of S for every* σ∈S*.*

Lemma 7 *Let* ϕ≠P⊆S*. Then, the following assertions are equivalent.*

(1) *P is a bi-ideal of S.*
(2) *The FBS characteristic function* $\overset{P}{\chi_{A}}$ *of P is an FBS bi-ideal over S.*

Proof It is straightforward. □

The following characterization of completely regular ordered semigroups, in terms of their principal bi-ideals, semiprimality of their bi-ideals and fuzzy semiprimality of their fuzzy bi-ideals, is due to Theorem 10 of N. Kehayopulu [36].

Lemma 8 *The following assertions are equivalent on S.*

(1) *S is completely regular.*
(2) *Every bi-ideal of S is semiprime.*
(3) *Every fuzzy bi-ideal of S is fuzzy semiprime.*
(4) $a\in B(a^{2})$ *for every* a∈S*.*
(5) $B(a)=B(a^{2})$ *for every* a∈S*.*

Now, in the following, we will give a characterization of completely regular ordered semigroups by replacing fuzzy semiprimality of their bi-ideals by FBS semiprimality of their bi-ideals in Lemma 8. Thus, we establish the following theorem:

Theorem 7 *The following assertions are equivalent on S.*

(1) *S is completely regular.*
(2) *Every bi-ideal of S is semiprime.*
(3) *Every FBS bi-ideal* $\lambda_{A}$ *over S is FBS semiprime.*
(4) $a\in B(a^{2})$ *for every* a∈S*.*
(5) $B(a)=B(a^{2})$ *for every* a∈S*.*

Proof First, assume that *S* is completely regular. In order to prove that (2) holds, let *B* be a bi-ideal of *S*. Suppose that a∈S such that $a^{2}\in B$. Since *S* is completely regular, thus, by Lemma 3, we have

$$a\in \left(a^{2}Sa^{2}\right]\subseteq \left(BSB\right]\subseteq \left(B\right]=B.$$

Thus *B* is semiprime and that Condition (1) implies (2). Now, assume that (2) holds. In order to prove that (3) holds, let $\lambda_{A}$ be an FBS bi-ideal over *S* and a∈S. By Lemma 6, the set $\left(a^{2}Sa^{2}\right]$ is a bi-ideal of *S*. Then, by the hypothesis, $\left(a^{2}Sa^{2}\right]$ is semiprime. Moreover, we have

$${\left(a^{4}\right)}^{2}=a^{8}\in \left(a^{2}Sa^{2}\right],$$

$${\left(a^{2}\right)}^{2}=a^{4}\in \left(a^{2}Sa^{2}\right],$$

$$a^{2}\in \left(a^{2}Sa^{2}\right],$$

which implies $a\in \left(a^{2}Sa^{2}\right]$. This further implies that $a\leq a^{2}xa^{2}$, for some x∈S. Then, since $\lambda_{A}$ is an FBS bi-ideal over *S*, thus we have, for all ε∈A,

$$\overset{+}{\lambda }(\varepsilon )(a)\geq \overset{+}{\lambda }(\varepsilon )(a^{2}xa^{2})\geq \min\{\overset{+}{\lambda }(\varepsilon )(a^{2}),\overset{+}{\lambda }(\varepsilon )(a^{2})\}=\overset{+}{\lambda }(\varepsilon )(a^{2}),$$

and

$$\overset{-}{\lambda }(\varepsilon )(a)\leq \overset{-}{\lambda_{{}_{A}}}(\varepsilon )(a^{2}xa^{2})\leq \max\{\overset{-}{\lambda }(\varepsilon )(a^{2}),\overset{-}{\lambda }(\varepsilon )(a^{2})\}=\overset{-}{\lambda }(\varepsilon )(a^{2}).$$

Therefore, $\lambda_{A}$ is FBS semiprime and that (2) implies (3). Next, assume that (3) holds. Let ε∈A and a∈S. Let's consider the bi-ideal $B(a^{2})=\left(a^{2}\cup a^{2}Sa^{2}\right]$ of *S* that is generated by $a^{2}$. Then, by Lemma 7, the FBS characteristic function $\overset{B(a^{2})}{\chi_{{}_{A}}}$ of $B(a^{2})$ is an FBS bi-ideal over *S*. Then, by the hypothesis, $\overset{B(a^{2})}{\chi_{{}_{A}}}$ is FBS semiprime. So, we have

$${\overset{+}{\chi }}_{{}_{B(a^{2})}}(\varepsilon )(a)\geq {\overset{+}{\chi }}_{{}_{B(a^{2})}}(\varepsilon )(a^{2}),\;\;\;\;\;{\overset{-}{\chi }}_{{}_{B(a^{2})}}(\varepsilon )(a)\leq {\overset{-}{\chi }}_{{}_{B(a^{2})}}(\varepsilon )(a^{2}),$$

and, since $a^{2}\in B(a^{2})$, we have

$${\overset{+}{\chi }}_{{}_{B(a^{2})}}(\varepsilon )(a^{2})=1,\;\;\;\;{\overset{-}{\chi }}_{{}_{B(a^{2})}}(\varepsilon )(a^{2})=0.$$

Therefore, it follows that

$${\overset{+}{\chi }}_{{}_{B(a^{2})}}(\varepsilon )(a)=1,\;\;\;\;{\overset{-}{\chi }}_{{}_{B(a^{2})}}(\varepsilon )(a)=0,$$

which implies that $a\in B(a^{2})$ and that (3) implies (4). Further, assume that (4) holds and a∈S. Then, by the hypothesis, we have

$$a\in B(a)\subseteq B(a^{2})=\left(a^{2}\cup a^{2}Sa^{2}\right].$$

Next, we have

$$a^{2}\in \left(a^{2}\cup a^{2}Sa^{2}\right]\left(a\right]\subseteq \left(a^{2}\cup a^{2}Sa^{2})a\right]=\left(a^{3}\cup a^{2}Sa^{3}\right]\subseteq \left(aSa\right]\subseteq \left(a\cup aSa\right]=B(a),$$

which implies that $B(a^{2})\subseteq B(a)$. Thus, we obtain $B(a)=B(a^{2})$ and that (4) implies (5). Finally, assume that (5) holds and a∈S. So, by the hypothesis, we have

$$a\in B(a)\subseteq B(a^{2})=\left(a^{2}\cup a^{2}Sa^{2}\right].$$

If $a\leq a^{2}$, then

$$aa\leq a^{2}a^{2}=aaa^{2}\leq a^{2}aa^{2}.$$

Replacing *a* by *x*, we obtain $a\leq a^{2}xa^{2}$. Thus, *S* is completely regular and that (5) implies (1). Thus the theorem follows. □

## 5 The Cartesian product of fuzzy bipolar soft semiprime (resp., prime) ideals over ordered semigroups

In this section, we consider the Cartesian product of two FBS semiprime (resp., prime) ideals over *S*. We show that the Cartesian product of two FBS semiprime (resp., prime) ideals over *S* is an FBS semiprime (resp., prime) ideal over S×S.

Definition 12 Let $\lambda_{A}$ and $\delta_{A}$ be FBS sets over *S*, and

g:A×A→f(A)×f(A)

be an injective mapping defined by g(α,β)=(f(α),f(β)), for all (α,β) in A×A. Then, the Cartesian product of $\lambda_{A}$ and $\delta_{A}$ is an FBS set $\gamma_{\Lambda }$ over S×S, where Λ=A×A, which is defined in terms of its fuzzy approximate functions as follows:

$$\overset{+}{\gamma }(\alpha ,\beta )=\overset{+}{\lambda }(\alpha )\wedge \overset{+}{\delta }(\beta ),$$

and

$$\overset{-}{\gamma }(\alpha ,\beta )=\overset{-}{\lambda }(\alpha )\vee \overset{-}{\delta }(\beta ),$$

for all (α,β)∈Λ. We denote $\gamma_{\Lambda }=\lambda_{A}\times \delta_{A}$, where $\overset{+}{\gamma }=\overset{+}{\lambda }\times \overset{+}{\delta }$ and $\overset{-}{\gamma }=\overset{-}{\lambda }\times \overset{-}{\delta }$. Here, the symbols ∧ and ∨ respectively represent fuzzy intersection and fuzzy union. Further, we note that

$$\overset{+}{\gamma }(\alpha ,\beta )((x,y))=\min\{\overset{+}{\lambda }(\alpha )(x),\overset{+}{\delta }(\beta )(y)\},$$

and

$$\overset{-}{\gamma }(\alpha ,\beta )((x,y))=\max\{\overset{-}{\lambda }(\alpha )(x),\overset{-}{\delta }(\beta )(y)\},$$

for all (α,β)∈Λ and (x,y)∈S×S.

Definition 13 Let $\lambda_{A}$ and $\delta_{A}$ be FBS sets over *S*. For each (α,β)∈A×A and the real numbers r∈(0,1], t∈[0,1), we denote by $\overset{\;\;\;\;\;\;\;\;\;\;\;\;\;\;\;(r,t)}{\left(\lambda_{A}\times \delta_{A})(\alpha ,\beta \right)}$ a subset of S×S defined as follows:

$$\overset{\;\;\;\;\;\;\;\;\;\;\;\;\;\;(r,t)}{\left(\lambda_{A}\times \delta_{A})(\alpha ,\beta \right)}=\{(x,y)\in S\times S:\overset{+}{\lambda }(\alpha )(x),\overset{+}{\delta }(\beta )(y)\geq r,\;\;\overset{-}{\lambda }(\alpha )(x),\overset{-}{\delta }(\beta )(y)\leq t\}.$$

For any (α,β)∈A×A, the subset $\overset{\;\;\;\;\;\;\;\;\;\;\;\;\;\;(r,t)}{\left(\lambda_{A}\times \delta_{A})(\alpha ,\beta \right)}$ of S×S is called an (r,t)-level subset of $\lambda_{A}\times \delta_{A}$.

Proposition 13 *Let* $\lambda_{A}$ *and* $\delta_{A}$ *be FBS sets over S, and let* r∈(0,1] *and* t∈[0,1)*. Then, we have*

$$\overset{\;\;\;\;\;\;\;\;\;\;\;\;\;\;(r,t)}{\left(\lambda_{A}\times \delta_{A})(\alpha ,\beta \right)}=\overset{\left(r,t\right)}{\lambda_{A}(\alpha )}\times \overset{\left(r,t\right)}{\lambda_{A}(\beta )}.$$

Proof It is straightforward. □

Lemma 9 *Let* $\lambda_{A}$ *and* $\delta_{A}$ *be FBS left (resp., right, two-sided) ideals over S. Then* $\lambda_{A}\times \delta_{A}$ *is an FBS left (resp., right, two-sided) ideal over* S×S*.*

Proof It is straightforward. □

Proposition 14 *Let* $\lambda_{A}$ *and* $\delta_{A}$ *be FBS left (resp., right, two-sided) ideals over S. Then, the* (r,t)*-level subset* $\overset{\;\;\;\;\;\;\;\;\;\;\;\;\;\;(r,t)}{\left(\lambda_{A}\times \delta_{A})(\alpha ,\beta \right)}(\neq \phi )$ *of* $\lambda_{A}\times \delta_{A}$ *is a left (resp., right, two-sided) ideal of* S×S*, for all* r∈(0,1]*,* t∈[0,1) *and* (α,β)∈A×A*.*

Proof Let $\gamma_{\Lambda }=\lambda_{A}\times \delta_{A}$, where Λ=A×A. Since $\lambda_{A}$ and $\delta_{A}$ are FBS left ideals over *S*, thus, by Lemma 9, we have $\gamma_{\Lambda }$ is an FBS left ideal over S×S. Now, for any r∈(0,1], t∈[0,1) and (α,β)∈Λ, we show that the (r,t)-level subset $\overset{(r,t)\;\;\;\;}{\gamma_{\Lambda }(\alpha ,\beta )}(\neq \phi )$ of $\gamma_{\Lambda }$ is a left ideal of S×S. For this, let (a,b)∈S×S and $(c,d)\in \overset{(r,t)\;\;\;\;}{\gamma_{\Lambda }(\alpha ,\beta )}$. Then, we have

$$\overset{+}{\gamma }(\alpha ,\beta )((a,b)(c,d))\geq \overset{+}{\gamma }(\alpha ,\beta )((c,d))\geq r,$$

and

$$\overset{-}{\gamma }(\alpha ,\beta )((a,b)(c,d))\leq \overset{-}{\gamma }(\alpha ,\beta )((c,d))\leq t.$$

This implies that $(a,b)(c,d)\in \overset{(r,t)\;\;\;\;}{\gamma_{\Lambda }(\alpha ,\beta )}$. Next, let (e,f) and (g,h) be any elements in S×S such that (e,f)≤(g,h) and that $(g,h)\in \overset{(r,t)\;\;\;\;}{\gamma_{\Lambda }(\alpha ,\beta )}$. Since $\gamma_{\Lambda }$ is a left ideal over S×S, we have

$$\overset{+}{\gamma }(\alpha ,\beta )((e,f))\geq \overset{+}{\gamma }(\alpha ,\beta )((g,h))\geq r,$$

and

$$\overset{-}{\gamma }(\alpha ,\beta )((e,f))\leq \overset{-}{\gamma }(\alpha ,\beta )((g,h))\leq t,$$

which implies that $(e,f)\in \overset{(r,t)\;\;\;\;}{\gamma_{\Lambda }(\alpha ,\beta )}$. Therefore, $\overset{(r,t)\;\;\;\;}{\gamma_{\Lambda }(\alpha ,\beta )}$ is a left ideal of S×S. The other parts of the proposition can be proved similarly. □

In the following theorem, we prove that the Cartesian product of two FBS semiprime ideals over *S* is an FBS semiprime ideal over S×S.

Theorem 8 *Let* $\lambda_{A}$ *and* $\delta_{A}$ *be FBS semiprime ideals over S. Then* $\lambda_{A}\times \delta_{A}$ *is an FBS semiprime ideal over* S×S*.*

Proof Let $\gamma_{\Lambda }=\lambda_{A}\times \delta_{A}$, where Λ=A×A. Then, by Lemma 9, we have $\gamma_{\Lambda }$ is an FBS ideal over S×S. To finish the proof, we just need to show that $\gamma_{\Lambda }$ is FBS semiprime. Since $\lambda_{A}$ and $\delta_{A}$ are FBS semiprime, thus, for all (a,b)∈S×S and (α,β)∈Λ, we have

$$\overset{+}{\gamma }(\alpha ,\beta )((a,b))=\min\{\overset{+}{\lambda }(\alpha )(a),\overset{+}{\delta }(\beta )(b)\}\geq \min\{\overset{+}{\lambda }(\alpha )(a^{2}),\overset{+}{\delta }(\beta )(b^{2})\}=\overset{+}{\gamma }(\alpha ,\beta )(a^{2},b^{2})=\overset{+}{\gamma }(\alpha ,\beta ){\left(a,b\right)}^{2}.$$

Similarly,

$$\overset{-}{\gamma }(\alpha ,\beta )((a,b))=\max\{\overset{-}{\lambda }(\alpha )(a),\overset{-}{\delta }(\beta )(b)\}\leq \max\{\overset{-}{\lambda }(\alpha )(a^{2}),\overset{-}{\delta }(\beta )(b^{2})\}=\overset{-}{\gamma }(\alpha ,\beta )(a^{2},b^{2})=\overset{-}{\gamma }(\alpha ,\beta ){\left(a,b\right)}^{2}.$$

Therefore, $\gamma_{\Lambda }$ is FBS semiprime. This completes the proof. □

Proposition 15 *Let* $\lambda_{A}$ *and* $\delta_{A}$ *be FBS semiprime ideals over S. Then, for all* r∈(0,1]*,* t∈[0,1) *and* (α,β)∈A×A*, the* (r,t)*-level subset* $\overset{\;\;\;\;\;\;\;\;\;\;\;\;\;\;(r,t)}{\left(\lambda_{A}\times \delta_{A})(\alpha ,\beta \right)}(\neq \phi )$ *of* $\lambda_{A}\times \delta_{A}$ *is a semiprime ideal of* S×S*.*

Proof Let $\gamma_{\Lambda }=\lambda_{A}\times \delta_{A}$, where Λ=A×A. Then, by Theorem 8, we have $\gamma_{\Lambda }$ is an FBS semiprime ideal over S×S. Further, by Proposition 14, we see that $\overset{(r,t)\;\;\;\;}{\gamma_{\Lambda }(\alpha ,\beta )}$ is an ideal of S×S for all r∈(0,1] and t∈[0,1). To finish the proof, we only need to show that $\overset{(r,t)\;\;\;\;}{\gamma_{\Lambda }(\alpha ,\beta )}$ is semiprime. For this, let (a,b)∈S×S such that ${\left(a,b\right)}^{2}\in \overset{\left(r,t\right)}{\gamma_{\Lambda }(\alpha ,\beta )}$. Then, for all (α,β)∈Λ, we have

$$\overset{+}{\gamma }(\alpha ,\beta )((a,b))\geq \overset{+}{\gamma }(\alpha ,\beta )({\left(a,b\right)}^{2})\geq r,$$

and

$$\overset{-}{\gamma }(\alpha ,\beta )((a,b))\leq \overset{-}{\gamma }(\alpha ,\beta )({\left(a,b\right)}^{2})\leq t.$$

This implies that $(a,b)\in \overset{(r,t)\;\;\;\;}{\gamma_{\Lambda }(\alpha ,\beta )}$. Therefore, $\overset{(r,t)\;\;\;\;}{\gamma_{\Lambda }(\alpha ,\beta )}$ is semiprime. This completes the proof. □

The following theorem characterizes the FBS primality of two FBS sets $\lambda_{A}$ and $\delta_{A}$ over *S* by the FBS primality of their Cartesian product $\lambda_{A}\times \delta_{A}$ over S×S.

Theorem 9 *Let* $\lambda_{A}$ *and* $\delta_{A}$ *be FBS prime ideals over S. Then* $\lambda_{A}\times \delta_{A}$ *is an FBS prime ideal over* S×S*.*

Proof Let $\gamma_{\Lambda }=\lambda_{A}\times \delta_{A}$, where Λ=A×A. Then $\gamma_{\Lambda }$ is, by Lemma 9, an FBS ideal over S×S. To finish the proof, we just need to show that $\gamma_{\Lambda }$ is FBS prime. For this, let (a,b),(c,d)∈S×S and (α,β)∈Λ. Then, since $\lambda_{A}$ and $\delta_{A}$ are FBS prime ideals over *S*, we have

$$\overset{+}{\gamma }(\alpha ,\beta )((a,b)(c,d))=\overset{+}{\gamma }(\alpha ,\beta )((ac,bd))=\min[\overset{+}{\lambda }(\alpha )(ac),\overset{+}{\delta }(\beta )(bd)]=\min[\max(\overset{+}{\lambda }(\alpha )(a),\overset{+}{\lambda }(\alpha )(c)),\max(\overset{+}{\delta }(\beta )(b),\overset{+}{\delta }(\beta )(d))]=\max[\min(\overset{+}{\lambda }(\alpha )(a),\overset{+}{\delta }(\beta )(b)),\min(\overset{+}{\lambda }(\alpha )(c),\overset{+}{\delta }(\beta )(d))]=\max[\overset{+}{\gamma }(\alpha ,\beta )((a,b)),\overset{+}{\gamma }(\alpha ,\beta )((c,d))].$$

Similarly,

$$\overset{-}{\gamma }(\alpha ,\beta )((a,b)(c,d))=\overset{-}{\gamma }(\alpha ,\beta )((ac,bd))=\max[\overset{-}{\lambda }(\alpha )(ac),\overset{-}{\delta }(\beta )(bd)]=\max[\min(\overset{-}{\lambda }(\alpha )(a),\overset{-}{\lambda }(\alpha )(c)),\min(\overset{-}{\delta }(\beta )(b),\overset{-}{\delta }(\beta )(d))]=\min[\max(\overset{-}{\lambda }(\alpha )(a),\overset{-}{\delta }(\beta )(b)),\max(\overset{-}{\lambda }(\alpha )(c),\overset{-}{\delta }(\beta )(d))]=\min[\overset{-}{\gamma }(\alpha ,\beta )((a,b)),\overset{-}{\gamma }(\alpha ,\beta )((c,d))].$$

Therefore, $\gamma_{\Lambda }$ is FBS prime. This completes the proof. □

As an explanation of Theorem 9, we present the following example.

Example 7 Consider the ordered semigroup S={a,b,c,d} with the multiplication “⋅” and the order relation “≤” given below:

**Table:** 

⋅	*a*	*b*	*c*	*d*
*a*	*b*	*b*	*a*	*a*
*b*	*b*	*b*	*b*	*b*
*c*	*a*	*b*	*c*	*c*
*d*	*a*	*b*	*c*	*c*

≤={(a,a),(a,b),(b,b),(c,c),(d,d)}.

Suppose $A=E=\{\varepsilon_{1},\varepsilon_{2}\}$ be a set of parameters and f:A→A be an identity function. Let $\lambda_{A}$ and $\delta_{A}$ be FBS sets over *S* that are defined, for all x∈S, as follows:

$$\overset{+}{\lambda }(\varepsilon_{1})(x)=\left\{ \begin{array}{cc} 0.5 & \text{if }x\in \{a,b\},\\ 0.3 & \text{if }x\in \{c,d\}, \end{array}\right.$$

$$\overset{+}{\lambda }(\varepsilon_{2})(x)=\left\{ \begin{array}{cc} 0.6 & \text{if }x\in \{a,b\},\\ 0.4 & \text{if }x\in \{c,d\}, \end{array}\right.$$

$$\overset{-}{\lambda }(\varepsilon_{1})(x)=\left\{ \begin{array}{cc} 0.4 & \text{if }x\in \{a,b\},\\ 0.6 & \text{if }x\in \{c,d\}, \end{array}\right.$$

$$\overset{-}{\lambda }(\varepsilon_{2})(x)=\left\{ \begin{array}{cc} 0.3 & \text{if }x\in \{a,b\},\\ 0.5 & \text{if }x\in \{c,d\}, \end{array}\right.$$

and

$$\overset{+}{\delta }(\varepsilon_{1})(x)=\left\{ \begin{array}{cc} 0.4 & \text{if }x\in \{a,b\},\\ 0.2 & \text{if }x\in \{c,d\}, \end{array}\right.$$

$$\overset{+}{\delta }(\varepsilon_{2})(x)=\left\{ \begin{array}{cc} 0.5 & \text{if }x\in \{a,b\},\\ 0.4 & \text{if }x\in \{c,d\}, \end{array}\right.$$

$$\overset{-}{\delta }(\varepsilon_{1})(x)=\left\{ \begin{array}{cc} 0.4 & \text{if }x\in \{a,b\},\\ 0.5 & \text{if }x\in \{c,d\}, \end{array}\right.$$

$$\overset{-}{\delta }(\varepsilon_{2})(x)=\left\{ \begin{array}{cc} 0.3 & \text{if }x\in \{a,b\},\\ 0.5 & \text{if }x\in \{c,d\}. \end{array}\right.$$

Clearly $\lambda_{A}$ and $\delta_{A}$ are FBS prime ideals over *S*.

Now, let $\gamma_{\Lambda }=\lambda_{A}\times \delta_{A}$, where Λ=A×A. Further, let g:Λ→f(A)×f(A) be an injective function such that $g(\varepsilon_{i},\varepsilon_{j})=(f(\varepsilon_{i}),f(\varepsilon_{j}))$ for all $\varepsilon_{i}$, $\varepsilon_{j}\in \Lambda$, where i,j=1,2. Then $\gamma_{\Lambda }$ can be defined as follows:

$$\overset{+}{\gamma }(\varepsilon_{1},\varepsilon_{1})((a,a))=0.4,\;\;\;\;\overset{+}{\gamma }(\varepsilon_{1},\varepsilon_{1})((a,b))=0.4,\;\;\;\;\overset{+}{\gamma }(\varepsilon_{1},\varepsilon_{1})((a,c))=0.2,$$

$$\overset{+}{\gamma }(\varepsilon_{1},\varepsilon_{1})((a,d))=0.2,\;\overset{+}{\gamma }(\varepsilon_{1},\varepsilon_{1})((b,a))=0.4,\;\;\;\;\overset{+}{\gamma }(\varepsilon_{1},\varepsilon_{1})((b,b))=0.4,$$

$$\overset{+}{\gamma }(\varepsilon_{1},\varepsilon_{1})((b,c))=0.2,\;\;\;\;\overset{+}{\gamma }(\varepsilon_{1},\varepsilon_{1})((b,d))=0.2,\;\;\;\;\overset{+}{\gamma }(\varepsilon_{1},\varepsilon_{1})((c,a))=0.3,$$

$$\overset{+}{\gamma }(\varepsilon_{1},\varepsilon_{1})((c,b))=0.3,\;\;\;\;\overset{+}{\gamma }(\varepsilon_{1},\varepsilon_{1})((c,c))=0.2,\;\;\;\;\overset{+}{\gamma }(\varepsilon_{1},\varepsilon_{1})((c,d))=0.2,$$

$$\overset{+}{\gamma }(\varepsilon_{1},\varepsilon_{1})((d,a))=0.3,\;\;\;\;\overset{+}{\gamma }(\varepsilon_{1},\varepsilon_{1})((d,b))=0.3,\;\;\;\;\overset{+}{\gamma }(\varepsilon_{1},\varepsilon_{1})((d,c))=0.2,$$

$$\overset{+}{\gamma }(\varepsilon_{1},\varepsilon_{1})((d,d))=0.2,\;\;\;\;\overset{+}{\gamma }(\varepsilon_{1},\varepsilon_{2})((a,a))=0.5,\;\;\;\;\overset{+}{\gamma }(\varepsilon_{1},\varepsilon_{2})((a,b))=0.5,$$

$$\overset{+}{\gamma }(\varepsilon_{1},\varepsilon_{2})((a,c))=0.4,\;\;\;\;\overset{+}{\gamma }(\varepsilon_{1},\varepsilon_{2})((a,d))=0.4,\;\;\;\;\overset{+}{\gamma }(\varepsilon_{1},\varepsilon_{2})((b,a))=0.5,$$

$$\overset{+}{\gamma }(\varepsilon_{1},\varepsilon_{2})((b,b))=0.5,\;\;\;\;\overset{+}{\gamma }(\varepsilon_{1},\varepsilon_{2})((b,c))=0.4,\;\;\;\;\overset{+}{\gamma }(\varepsilon_{1},\varepsilon_{2})((b,d))=0.4,$$

$$\overset{+}{\gamma }(\varepsilon_{1},\varepsilon_{2})((c,a))=0.3,\;\;\;\;\overset{+}{\gamma }(\varepsilon_{1},\varepsilon_{2})((c,b))=0.3,\;\;\;\;\overset{+}{\gamma }(\varepsilon_{1},\varepsilon_{2})((c,c))=0.3,$$

$$\overset{+}{\gamma }(\varepsilon_{1},\varepsilon_{2})((c,d))=0.3,\;\;\;\;\overset{+}{\gamma }(\varepsilon_{1},\varepsilon_{2})((d,a))=0.3,\;\;\;\;\overset{+}{\gamma }(\varepsilon_{1},\varepsilon_{2})((d,b))=0.3,$$

$$\overset{+}{\gamma }(\varepsilon_{1},\varepsilon_{2})((d,c))=0.3,\;\;\;\;\overset{+}{\gamma }(\varepsilon_{1},\varepsilon_{2})((d,d))=0.3,\;\;\;\;\overset{+}{\gamma }(\varepsilon_{2},\varepsilon_{1})((a,a))=0.4,$$

$$\overset{+}{\gamma }(\varepsilon_{2},\varepsilon_{1})((a,b))=0.4,\;\;\;\;\overset{+}{\gamma }(\varepsilon_{2},\varepsilon_{1})((a,c))=0.2,\;\;\;\;\overset{+}{\gamma }(\varepsilon_{2},\varepsilon_{1})((a,d))=0.2,$$

$$\overset{+}{\gamma }(\varepsilon_{2},\varepsilon_{1})((b,a))=0.4,\;\;\;\;\overset{+}{\gamma }(\varepsilon_{2},\varepsilon_{1})((b,b))=0.4,\;\;\;\;\overset{+}{\gamma }(\varepsilon_{2},\varepsilon_{1})((b,c))=0.2,$$

$$\overset{+}{\gamma }(\varepsilon_{2},\varepsilon_{1})((b,d))=0.2,\;\;\;\;\overset{+}{\gamma }(\varepsilon_{2},\varepsilon_{1})((c,a))=0.4,\;\;\;\;\overset{+}{\gamma }(\varepsilon_{2},\varepsilon_{1})((c,b))=0.4,$$

$$\overset{+}{\gamma }(\varepsilon_{2},\varepsilon_{1})((c,c))=0.2,\;\;\;\;\overset{+}{\gamma }(\varepsilon_{2},\varepsilon_{1})((c,d))=0.2,\;\;\;\;\overset{+}{\gamma }(\varepsilon_{2},\varepsilon_{1})((d,a))=0.4,$$

$$\overset{+}{\gamma }(\varepsilon_{2},\varepsilon_{1})((d,b))=0.4,\;\;\;\;\overset{+}{\gamma }(\varepsilon_{2},\varepsilon_{1})((d,c))=0.2,\;\;\;\;\overset{+}{\gamma }(\varepsilon_{2},\varepsilon_{1})((d,d))=0.2,$$

$$\overset{+}{\gamma }(\varepsilon_{2},\varepsilon_{2})((a,a))=0.5,\;\;\;\;\overset{+}{\gamma }(\varepsilon_{2},\varepsilon_{2})((a,b))=0.5,\;\;\;\;\overset{+}{\gamma }(\varepsilon_{2},\varepsilon_{2})((a,c))=0.4,$$

$$\overset{+}{\gamma }(\varepsilon_{2},\varepsilon_{2})((a,d))=0.4,\;\;\;\;\overset{+}{\gamma }(\varepsilon_{2},\varepsilon_{2})((b,a))=0.5,\;\;\;\;\overset{+}{\gamma }(\varepsilon_{2},\varepsilon_{2})((b,b))=0.5,$$

$$\overset{+}{\gamma }(\varepsilon_{2},\varepsilon_{2})((b,c))=0.4,\;\;\;\;\overset{+}{\gamma }(\varepsilon_{2},\varepsilon_{2})((b,d))=0.4,\;\;\;\;\overset{+}{\gamma }(\varepsilon_{2},\varepsilon_{2})((c,a))=0.4,$$

$$\overset{+}{\gamma }(\varepsilon_{2},\varepsilon_{2})((c,b))=0.4,\;\;\;\;\overset{+}{\gamma }(\varepsilon_{2},\varepsilon_{2})((c,c))=0.4,\;\;\;\;\overset{+}{\gamma }(\varepsilon_{2},\varepsilon_{2})((c,d))=0.4,$$

$$\overset{+}{\gamma }(\varepsilon_{2},\varepsilon_{2})((d,a))=0.4,\;\;\;\;\overset{+}{\gamma }(\varepsilon_{2},\varepsilon_{2})((d,b))=0.4,\;\;\;\;\overset{+}{\gamma }(\varepsilon_{2},\varepsilon_{2})((d,c))=0.4,$$

$$\overset{+}{\gamma }(\varepsilon_{2},\varepsilon_{2})((d,d))=0.4.$$

Similarly,

$$\overset{-}{\gamma }(\varepsilon_{1},\varepsilon_{1})((a,a))=0.4,\;\;\;\;\overset{-}{\gamma }(\varepsilon_{1},\varepsilon_{1})((a,b))=0.4,\;\;\;\;\overset{-}{\gamma }(\varepsilon_{1},\varepsilon_{1})((a,c))=0.5,$$

$$\overset{-}{\gamma }(\varepsilon_{1},\varepsilon_{1})((a,d))=0.5,\;\;\;\;\overset{-}{\gamma }(\varepsilon_{1},\varepsilon_{1})((b,a))=0.4,\;\;\;\;\overset{-}{\gamma }(\varepsilon_{1},\varepsilon_{1})((b,b))=0.4,$$

$$\overset{-}{\gamma }(\varepsilon_{1},\varepsilon_{1})((b,c))=0.5,\;\;\;\;\overset{-}{\gamma }(\varepsilon_{1},\varepsilon_{1})((b,d))=0.5,\;\;\;\;\overset{-}{\gamma }(\varepsilon_{1},\varepsilon_{1})((c,a))=0.6,$$

$$\overset{-}{\gamma }(\varepsilon_{1},\varepsilon_{1})((c,b))=0.6,\;\;\;\;\overset{-}{\gamma }(\varepsilon_{1},\varepsilon_{1})((c,c))=0.6,\;\;\;\;\overset{-}{\gamma }(\varepsilon_{1},\varepsilon_{1})((c,d))=0.6,$$

$$\overset{-}{\gamma }(\varepsilon_{1},\varepsilon_{1})((d,a))=0.6,\;\;\;\;\overset{-}{\gamma }(\varepsilon_{1},\varepsilon_{1})((d,b))=0.6,\;\;\;\;\overset{-}{\gamma }(\varepsilon_{1},\varepsilon_{1})((d,c))=0.6,$$

$$\overset{-}{\gamma }(\varepsilon_{1},\varepsilon_{1})((d,d))=0.6,\;\;\;\;\overset{-}{\gamma }(\varepsilon_{1},\varepsilon_{2})((a,a))=0.4,\;\;\;\;\overset{-}{\gamma }(\varepsilon_{1},\varepsilon_{2})((a,b))=0.4,$$

$$\overset{-}{\gamma }(\varepsilon_{1},\varepsilon_{2})((a,c))=0.5,\;\;\;\;\overset{-}{\gamma }(\varepsilon_{1},\varepsilon_{2})((a,d))=0.5,\;\;\;\;\overset{-}{\gamma }(\varepsilon_{1},\varepsilon_{2})((b,a))=0.4,$$

$$\overset{-}{\gamma }(\varepsilon_{1},\varepsilon_{2})((b,b))=0.4,\;\;\;\;\overset{-}{\gamma }(\varepsilon_{1},\varepsilon_{2})((b,c))=0.5,\;\;\;\;\overset{-}{\gamma }(\varepsilon_{1},\varepsilon_{2})((b,d))=0.5,$$

$$\overset{-}{\gamma }(\varepsilon_{1},\varepsilon_{2})((c,a))=0.6,\;\;\;\;\overset{-}{\gamma }(\varepsilon_{1},\varepsilon_{2})((c,b))=0.6,\;\;\;\;\overset{-}{\gamma }(\varepsilon_{1},\varepsilon_{2})((c,c))=0.6,$$

$$\overset{-}{\gamma }(\varepsilon_{1},\varepsilon_{2})((c,d))=0.6,\;\;\;\;\overset{-}{\gamma }(\varepsilon_{1},\varepsilon_{2})((d,a))=0.6,\;\;\;\;\overset{-}{\gamma }(\varepsilon_{1},\varepsilon_{2})((d,b))=0.6,$$

$$\overset{-}{\gamma }(\varepsilon_{1},\varepsilon_{2})((d,c))=0.6,\;\;\;\;\overset{-}{\gamma }(\varepsilon_{1},\varepsilon_{2})((d,d))=0.6,\;\;\;\;\overset{-}{\gamma }(\varepsilon_{2},\varepsilon_{1})((a,a))=0.4,$$

$$\overset{-}{\gamma }(\varepsilon_{2},\varepsilon_{1})((a,b))=0.4,\;\;\;\;\overset{-}{\gamma }(\varepsilon_{2},\varepsilon_{1})((a,c))=0.5,\;\;\;\;\overset{-}{\gamma }(\varepsilon_{2},\varepsilon_{1})((a,d))=0.5,$$

$$\overset{-}{\gamma }(\varepsilon_{2},\varepsilon_{1})((b,a))=0.4,\;\;\;\;\overset{-}{\gamma }(\varepsilon_{2},\varepsilon_{1})((b,b))=0.4,\;\;\;\;\overset{-}{\gamma }(\varepsilon_{2},\varepsilon_{1})((b,c))=0.5,$$

$$\overset{-}{\gamma }(\varepsilon_{2},\varepsilon_{1})((b,d))=0.5,\;\;\;\;\overset{-}{\gamma }(\varepsilon_{2},\varepsilon_{1})((c,a))=0.5,\;\;\;\;\overset{-}{\gamma }(\varepsilon_{2},\varepsilon_{1})((c,b))=0.5,$$

$$\overset{-}{\gamma }(\varepsilon_{2},\varepsilon_{1})((c,c))=0.5,\;\;\;\;\overset{-}{\gamma }(\varepsilon_{2},\varepsilon_{1})((c,d))=0.5,\;\;\;\;\overset{-}{\gamma }(\varepsilon_{2},\varepsilon_{1})((d,a))=0.5,$$

$$\overset{-}{\gamma }(\varepsilon_{2},\varepsilon_{1})((d,b))=0.5,\;\;\;\;\overset{-}{\gamma }(\varepsilon_{2},\varepsilon_{1})((d,c))=0.5,\;\;\;\;\overset{-}{\gamma }(\varepsilon_{2},\varepsilon_{1})((d,d))=0.5,$$

$$\overset{-}{\gamma }(\varepsilon_{2},\varepsilon_{2})((a,a))=0.3,\;\;\;\;\overset{-}{\gamma }(\varepsilon_{2},\varepsilon_{2})((a,b))=0.3,\;\;\;\;\overset{-}{\gamma }(\varepsilon_{2},\varepsilon_{2})((a,c))=0.5,$$

$$\overset{-}{\gamma }(\varepsilon_{2},\varepsilon_{2})((a,d))=0.5,\;\;\;\;\overset{-}{\gamma }(\varepsilon_{2},\varepsilon_{2})((b,a))=0.3,\;\;\;\;\overset{-}{\gamma }(\varepsilon_{2},\varepsilon_{2})((b,b))=0.3,$$

$$\overset{-}{\gamma }(\varepsilon_{2},\varepsilon_{2})((b,c))=0.5,\;\;\;\;\overset{-}{\gamma }(\varepsilon_{2},\varepsilon_{2})((b,d))=0.5,\;\;\;\;\overset{-}{\gamma }(\varepsilon_{2},\varepsilon_{2})((c,a))=0.5,$$

$$\overset{-}{\gamma }(\varepsilon_{2},\varepsilon_{2})((c,b))=0.5,\;\;\;\;\overset{-}{\gamma }(\varepsilon_{2},\varepsilon_{2})((c,c))=0.5,\;\;\;\;\overset{-}{\gamma }(\varepsilon_{2},\varepsilon_{2})((c,d))=0.5,$$

$$\overset{-}{\gamma }(\varepsilon_{2},\varepsilon_{2})((d,a))=0.5,\;\;\;\;\overset{-}{\gamma }(\varepsilon_{2},\varepsilon_{2})((d,b))=0.5,\;\;\;\;\overset{-}{\gamma }(\varepsilon_{2},\varepsilon_{2})((d,c))=0.5,$$

$$\overset{-}{\gamma }(\varepsilon_{2},\varepsilon_{2})((d,d))=0.5.$$

One can check that $\gamma_{\Lambda }$ is an FBS ideal over S×S. Likewise, the FBS semiprimality of $\gamma_{\Lambda }$ can be verified. For example, for any elements (a,b), (c,d)∈S×S and $(\varepsilon_{1},\varepsilon_{2})\in \Lambda$, we have

$$\overset{+}{\gamma }(\varepsilon_{1},\varepsilon_{2})((a,b)(c,d))=\overset{+}{\gamma }(\varepsilon_{1},\varepsilon_{2})((ac,bd))=\min\{\overset{+}{\lambda }(\varepsilon_{1})(ac),\overset{+}{\delta }(\varepsilon_{2})(bd)\}=\min\{\overset{+}{\lambda }(\varepsilon_{1})(a),\overset{+}{\delta }(\varepsilon_{2})(b)\}=0.5.$$

Moreover, we have

$$\overset{+}{\gamma }(\varepsilon_{1},\varepsilon_{2})((a,b))=\min\{\overset{+}{\lambda }(\varepsilon_{1})(a),\overset{+}{\delta }(\varepsilon_{2})(b)\}=\min\{0.5,0.5\}=0.5,$$

and

$$\overset{+}{\gamma }(\varepsilon_{1},\varepsilon_{2})((c,d))=\min\{\overset{+}{\lambda }(\varepsilon_{1})(c),\overset{+}{\delta }(\varepsilon_{2})(d)\}=\min\{0.3,0.4\}=0.3.$$

Thus, it follows that

$$\overset{+}{\gamma }(\varepsilon_{1},\varepsilon_{2})((a,b)(c,d))=\max\{\overset{+}{\gamma }(\varepsilon_{1},\varepsilon_{2})((a,b)),\overset{+}{\gamma }(\varepsilon_{1},\varepsilon_{2})((c,d))\}.$$

Similarly,

$$\overset{-}{\gamma }(\varepsilon_{1},\varepsilon_{2})((a,b)(c,d))=\min\{\overset{-}{\gamma }(\varepsilon_{1},\varepsilon_{2})((a,b)),\overset{-}{\gamma }(\varepsilon_{1},\varepsilon_{2})((c,d))\}.$$

In the following proposition, we characterize the Cartesian product $\lambda_{A}\times \delta_{A}$ (over S×S) of two FBS prime ideals $\lambda_{A}$ and $\delta_{A}$ (over *S*) by its (r,t)-level set.

Proposition 16 *Let* $\lambda_{A}$ *and* $\delta_{A}$ *be FBS prime ideals over S. Then, for all* r∈(0,1]*,* t∈[0,1) *and* (α,β)∈A×A*, the* (r,t)*-level subset* $\overset{\;\;\;\;\;\;\;\;\;\;\;(r,t)}{\left(\lambda_{A}\times \delta_{A})(\alpha ,\beta \right)}$ *(if it is nonempty) of* $\lambda_{A}\times \delta_{A}$ *is a prime ideal of* S×S*.*

Proof It is straightforward. □

## 6 Conclusion

In this article, the notion of FBS semiprimality in ordered semigroups is introduced. Some properties of the concept are investigated on left (resp., right, intra-, completely) regular and Archimedean ordered semigroups. It is revealed that if *S* is completely regular, then every FBS ideal $\lambda_{A}$ over *S* is FBS semiprime. Furthermore, the Cartesian product of FBS semiprime (resp., prime) ideals over ordered semigroups is studied. It is exposed that the Cartesian product $\lambda_{A}\times \delta_{A}$ of FBS semiprime (resp., prime) ideals $\lambda_{A}$ and $\delta_{A}$ over *S* is an FBS semiprime (resp., prime) ideal over S×S. It is worth to mention that the concept of FBS semiprimality can be extended to other classes of FBS ideals over ordered semigroups, for example, FBS interior and FBS quasi-ideals.

## Declarations

### Author contribution statement

Aziz-Ul-Hakim, H. Khan, I. Ahmad, K. Khan: Conceived and designed the analysis; Analyzed and interpreted the data; Contributed analysis tools or data; Wrote the paper.

### Funding statement

This research did not receive any specific grant from funding agencies in the public, commercial, or not-for-profit sectors.

### Data availability statement

No data was used for the research described in the article.

### Declaration of interests statement

The authors declare no conflict of interest.

### Additional information

No additional information is available for this paper.
